# Developing a genetic signature to predict drug response in ovarian cancer

**DOI:** 10.18632/oncotarget.23663

**Published:** 2017-12-26

**Authors:** Stephen Hyter, Jeff Hirst, Harsh Pathak, Ziyan Y. Pessetto, Devin C. Koestler, Rama Raghavan, Dong Pei, Andrew K. Godwin

**Affiliations:** ^1^ Department of Pathology and Laboratory Medicine, University of Kansas Medical Center, Kansas City, KS, USA; ^2^ University of Kansas Cancer Center, University of Kansas Medical Center, Kansas City, KS, USA; ^3^ Department of Biostatistics, University of Kansas Medical Center, Kansas City, KS, USA

**Keywords:** auranofin, AUY922, ovarian, gene signature, TCGA

## Abstract

There is a lack of personalized treatment options for women with recurrent platinum-resistant ovarian cancer. Outside of bevacizumab and a group of poly ADP-ribose polymerase inhibitors, few options are available to women that relapse. We propose that efficacious drug combinations can be determined via molecular characterization of ovarian tumors along with pre-established pharmacogenomic profiles of repurposed compounds. To that end, we selectively performed multiple two-drug combination treatments in ovarian cancer cell lines that included reactive oxygen species inducers and HSP90 inhibitors. This allowed us to select cell lines that exhibit disparate phenotypes of proliferative inhibition to a specific drug combination of auranofin and AUY922. We profiled altered mechanistic responses from these agents in both reactive oxygen species and HSP90 pathways, as well as investigated PRKCI and lncRNA expression in ovarian cancer cell line models. Generation of dual multi-gene panels implicated in resistance or sensitivity to this drug combination was produced using RNA sequencing data and the validity of the resistant signature was examined using high-density RT-qPCR. Finally, data mining for the prevalence of these signatures in a large-scale clinical study alluded to the prevalence of resistant genes in ovarian tumor biology. Our results demonstrate that high-throughput viability screens paired with reliable *in silico* data can promote the discovery of effective, personalized therapeutic options for a currently untreatable disease.

## INTRODUCTION

Drug repurposing circumvents the high costs and extended timeframes associated with drug discovery. It is a cost-effective approach to identifying and prioritizing novel therapeutic combinations for low prevalence yet highly lethal diseases such as epithelial ovarian cancer (EOC), the deadliest of the gynecological diseases. EOC is diagnosed in 225,000 women worldwide and results in more than 140,000 deaths annually [1]. Though primary tumors typically respond to frontline treatment, peritoneal dissemination of malignant cells resistant to taxanes and/or platinum-based compounds eventually result in recurrent disease. There are no common causative somatic gene mutations, outside of *TP53* alterations in serous adenocarcinoma and *KRAS, BRAF* and *PTEN* in mucinous, endometrioid, and low grade serous cancers, suggested to be associated with the pathogenesis of EOC [2]. Since there is not a dominant pathway to exploit across these genetically complex tumors, generalized EOC-specific targeted therapy has proven elusive and for the most part futile. Precision cancer medicine directed at distinct tumor vulnerabilities is imperative to make the vertical advancement in treatment in order to improve quality and duration of life for EOC patients. Consequently, there is a critical need to screen EOC tumors for specific genetic signatures to determine the most effective combinatorial treatment options on a case-by-case basis. Until then, chemotherapy resistance and drug toxicity will continue to hinder improvements in overall survival of patients with EOC.

Clinically relevant genetic signatures should both stratify patients into prognostic subtypes and predict chemotherapeutic response [3]. Relying solely on clinicopathologic markers to guide treatment decisions has proven ineffective mainly due to lack of knowledge of the inherent molecular vulnerabilities within individual tumors. The development of early prediction models initially utilized microarray technology to discern small gene sets predictive of treatment response [4–7]. However, as sequencing and gene silencing technologies continue to become commonplace, a more exact analysis of tumor biology is possible [8–10]. If solid tumors are truly heterogeneous diseases with assorted molecular features, hybrid signatures that take into account a combination of clinical and genetic characteristics allow oncologists the most informed criteria possible regarding therapeutic choices and risk of recurrence. The introduction of online publication compendiums and queryable databases offer a vast resource of data mining at little to no cost of access [11–14], this enables bioinformatical inquiry to become an integral component of modern research projects. Meta-analyses of multiple data sets, inconceivable before the advent of Internet technology, allows a global perspective on genetic relationships across a spectrum of tumor types [15–17]. The molecular assay PAM50 (and the clinical equivalent Prosigna^®)^ run on the NanoString nCounter^®^-platform is an example of an integrated breast cancer-subtyping platform developed for use in local laboratories [18, 19]. The Cancer Genome Atlas (TCGA) supplies both mutational and clinical data for a 33 tumor types, including ovarian serous adenocarcinoma [2], allowing researchers to identify molecular subtypes with survival correlations for EOC patients [20, 21]. Therefore, novel computational approaches identifying clinically relevant predictive signatures have the potential to advance EOC therapy in ways not conceivable through traditional basic science approaches [22, 23].

Auranofin (Ridaura^®^) is a gold complex originally approved as an antirheumatic agent that has emerged as a potential candidate for multiple repurposed therapies including neurodegenerative diseases, HIV/AIDS and microbial infections [24, 25]. Auranofin has an acceptable safety profile and has recently completed a Phase 2 study for treatment of chronic lymphocytic leukemia [26, 27]. Even though its specific anti-inflammatory mechanisms are not fully understood, auranofin also demonstrates several anticancer properties. Its most well studied mechanism of action is inhibition of thioredoxin reductase (TrxR) enzyme with subsequent induction of reactive oxygen species (ROS) [28, 29]. It has also been shown to mimic proteasomal inhibition by acting upon proteasome-associated deubiquitinases (DUBs) [30]. Other areas of research for the anti-growth activity of this heavy metal compound focus on inhibition of the STAT3 and NFκB pathways [31, 32]. When directed against EOC cell lines, additional mechanisms of action have been demonstrated. It was shown to be more potent than cisplatin in decreasing cell viability, especially in cells conditioned for cisplatin-resistance [33]. In addition, studies in ovarian cancer models have implicated auranofin as a potent activator of the FOXO3 tumor suppressor [34], as well as a selective inhibitor of oncogenic protein kinase C iota (PKCι) signaling [35]. The latter studies include a clinical trial evaluating the benefits of oral dosing of auranofin alongside CA-125 monitoring in asymptomatic ovarian cancer patients [36]. Auranofin has also been shown to be more effective in BRCA1-defective ovarian cells due to accumulations in unrepaired DNA damage [37]; however, this observation has not been confirmed clinically. Importantly, substantial evidence has demonstrated the synergistic enhancement of auranofin effects when used in combination with other compounds [28, 38–42].

The HSP90 chaperone protein utilizes an ATP-dependent mechanism to orchestrate various cellular functions, including the protection and activation of oncogenic client proteins [43]. Due to exploitation of its housekeeping functions by malignant cells, therapeutics directed at disabling HSP90 activity is a current area of drug discovery. Though early natural products induced severe toxicities, current synthetic analogs display acceptable safety profiles and are being investigated in the treatment of a broad spectrum of neoplasms [43]. Among these next-generation compounds are AUY922 (Vernalis) and ganetespib, both of which are resorcinol class compounds that inhibit the ATP-binding domain of HSP90 [44–46], though few investigations have evaluated their impact on ovarian tumor biology. One such study was a meta-analysis of siRNA screens indicating that degradation of HSP90 client proteins has the potential to sensitize EOC cell lines to additional targeted therapies. Moreover, exposure to ganetespib sensitized orthotopic ovarian xenografts to treatment with paclitaxel [47]. Although not currently being investigated in the treatment of EOC, studies have also demonstrated AUY922 efficacy in EOC models [48, 49].

As indicated, current methods of clinical intervention for EOC patients are dismal. Although the introduction of platinum and taxane-based therapy led to substantial improvements in patient survival, very little progress has occurred over the last few decades. In order to repurpose available FDA-cleared compounds based on pharmacogenomic profiles, we attempted to classify a phenotypic response to the combinatorial effects of auranofin and AUY922 through the use of transcriptional expression data. We selectively performed twelve different two-drug combination treatments in ten EOC cell lines and utilized RNA sequencing data to group expression signatures according to drug sensitivity or resistance. We show that combinations of auranofin (ROS inducer) and AUY922 (HSP90 antagonist) are highly potent towards a subset of EOC cells while others show inherent resistance. We have demonstrated that these lines exhibit dissimilar disruption of ROS homeostasis after auranofin treatment, while AUY922 activity displays potency across all lines. The *in silico* analyses identified two 23-gene panels correlated with either resistance or sensitivity to this combination and interrogation of these gene sets in the TCGA data set demonstrate a proclivity of tumors expressing genes related to the resistant panel more so than the sensitive panel. The goal of this project is to provide evidence for pre-treatment transcriptional profiling of clinical tumors in order to predict efficacy of novel drug combinations, as well as identify genetic markers involved in chemotherapeutic response by integrating high-throughput screening and *in silico* exploration.

## RESULTS

### Viability studies of FDA-approved drugs in EOC cell lines

**Figure 1:**
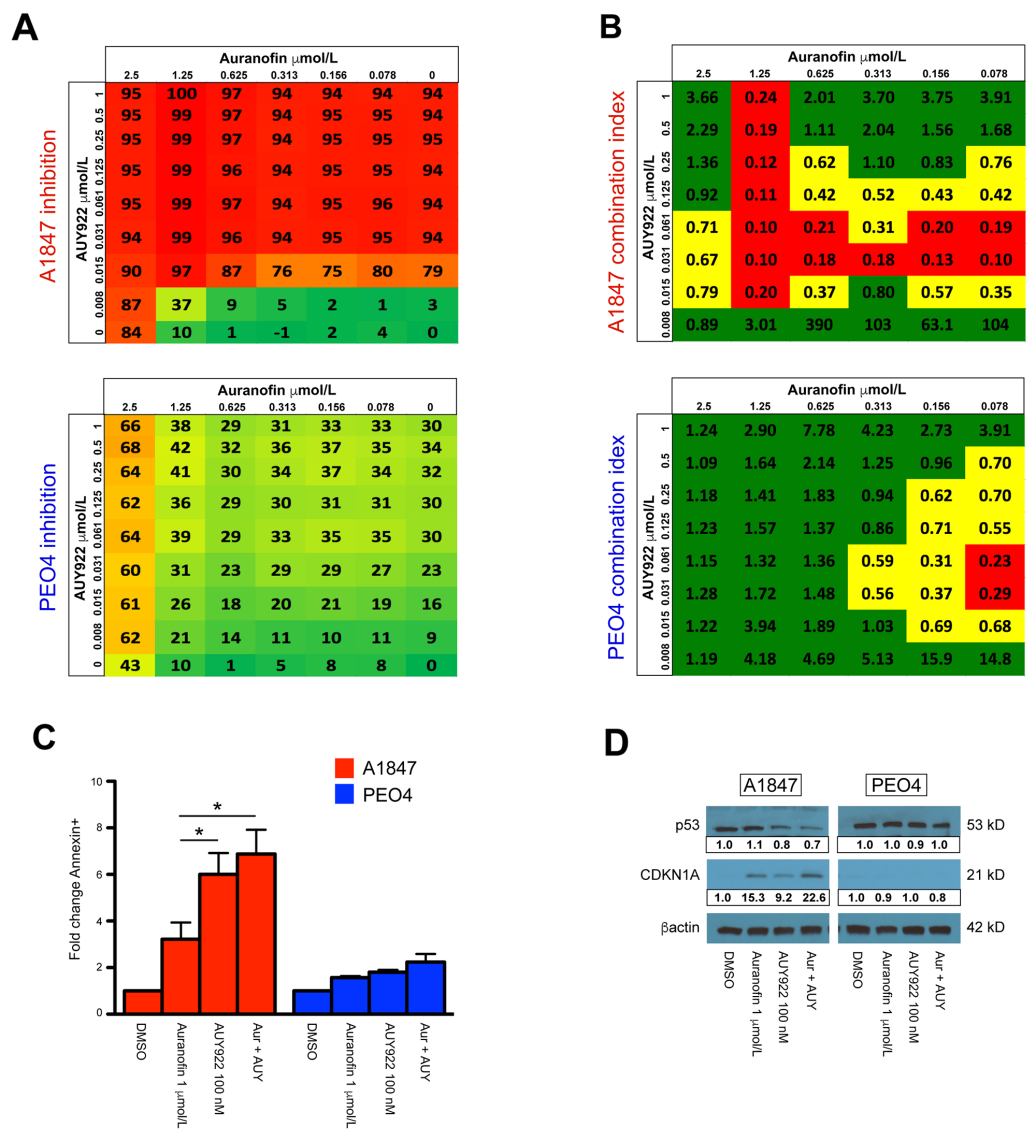
Combination analysis for auranofin and AUY922 in sensitive A1847 and resistant PEO4 cell lines (**A**) Color scale for percentage of viability inhibition is shown across 48 different drug combinations of auranofin and AUY922 for A1847 (upper) and PEO4 (lower) cell lines. (**B**) Dose response data was used to calculate the Combination Index (CI) values for auranofin and AUY922 combination treatment for A1847 (upper) and PEO4 (lower). Shown is the average calculated CI value ± standard error of the mean. CI value of >0.8 [green] indicates no synergy; CI = 0.3-0.8 [yellow] indicates synergistic effects; CI value of <0.3 [red] indicates strong synergistic effects. (**C**) Fold-change of A1847 and PEO4 cells positive for Annexin V staining 48 hours after incubation with indicated compounds, both alone and in combination (Aur + AUY). Data were quantified for fold-changes relative to vehicle treated cells and are presented as bar graphs showing average fold-change ± standard error of the mean, ^*^ = p < 0.05. (**D**) Protein expression of TP53 and CDKN1A 24 hours after incubation with indicated compounds, both alone and in combination (Aur + AUY). β-actin was used as a loading control and densitometric analysis of western blot data was performed using ImageJ software.

Utilizing a robotic screening assay in a 384-well format, we exposed ten EOC cell lines to twelve different two-drug combination treatments using FDA-approved drugs not currently in use as EOC therapies (Supplementary Table 1). Although many of the single agent and drug combinations showed robust inhibition of cell viability across all lines, we were interested in drug combinations that demonstrate disparate phenotypes across EOC cell lines. After analyzing the preliminary data, we chose to further investigate the combinatorial effects of auranofin (a reported ROS inducer) paired with either AUY922 or ganetespib (HSP90 inhibitors). This mechanistic combination was chosen due to the diverse effects we saw on viability among the panel of EOC lines, where some lines showed sensitivity to the combination (A1847, A2780, OVCAR8) while others displayed resistance (OVCAR4, PEO4, SKOV3) (Supplementary Figure 1A & 1B). In order to validate these results in a 96-well format, we first performed single agent viability screens using the three compounds to determine dose response curves of each cell line (Supplementary Figure 1C-1E). Initial investigations revealed that the two HSP90 inhibitors exhibited similar activities across the cell lines; therefore, we focused our efforts on a single combination of auranofin and AUY922. We then confirmed the preliminary high-throughput viability results to this drug combination in the two groups of EOC cell lines using a checkerboard design (Figure 1A & Supplementary Figure 2A). In further experiments, we utilized A1847 and PEO4 as representative cell lines for the sensitive and resistant groups, respectively.

To distinguish synergistic from additive effects of auranofin and AUY922, a combination index (CI) was calculated for each of the sensitive and resistant cell lines (Figure 1B & Supplementary Figure 2B). The CI model, originally developed by Chou *et al.* [50], is commonly used to assess drug interactions. Of the 48 total concentrations, auranofin and AUY922 are synergistic (<0.8 CI score) at 56% of the concentrations against A1847 cells compared to 27% for PEO4 cells. Moreover, this drug combination is highly synergistic (<0.3 CI score) for 29% of drug concentrations against A1847 cells versus only 4% for the PEO4 line (Figure 1B).

### Induction of apoptosis is increased in sensitive cells

Due to the synergistic effects seen in the sensitive EOC cell lines, we investigated if cellular viability was mediated through an apoptotic mechanism. To examine auranofin and AUY922-induced apoptosis, we evaluated Annexin V staining 48 hours after single agent and combinatorial treatment at the indicated concentrations (Figure 1C & Supplementary Figure 3A). After treatment, the fraction of Annexin V positive A1847 cells increased by an average of 3.2-fold in the auranofin group, 6.0-fold in the AUY922 group and 6.9-fold in the combination group relative to the vehicle group. Alternatively, in the resistant PEO4 cells the average induction of Annexin V staining was 1.6-fold in the auranofin group, 1.8-fold in the AUY922 and only 2.2-fold in the combination group relative to the vehicle group. We also evaluated p53 and CDKN1A protein levels after drug treatment (Figure 1D & Supplementary Figure 3B). Western blot analysis demonstrated strong p53 expression in A1847, A2780, OVCAR4 and PEO4 cells, yet undetectable in both OVCAR8 and SKOV3. Treatment of cells with auranofin for 24 hours did not alter p53 expression in any of the tested cell lines, though exposure to AUY922 alone or in combination with auranofin decreased expression of p53 in the sensitive A1847 and resistant OVCAR4 lines. A downstream effector of p53, the cyclin dependent kinase inhibitor CDKN1A, was increased in the sensitive A1847 after incubation with both auranofin and AUY922, as well as after combinatorial treatment of the two compounds. The resistant PEO4 line failed to demonstrate a marked increase in CDKN1A after any type of treatment, corroborating the decreased Annexin V staining seen post-treatment. Increased CDKN1A expression was also seen after single agent and combinatorial treatment in sensitive A2780 and resistant OVCAR4 cell lines, undetectable in OVCAR8 regardless of treatment and only increased after exposure to auranofin in SKOV3 cells (Supplementary Figure 3B).

### Auranofin-induced disruptions of reactive oxygen pathways

**Figure 2:**
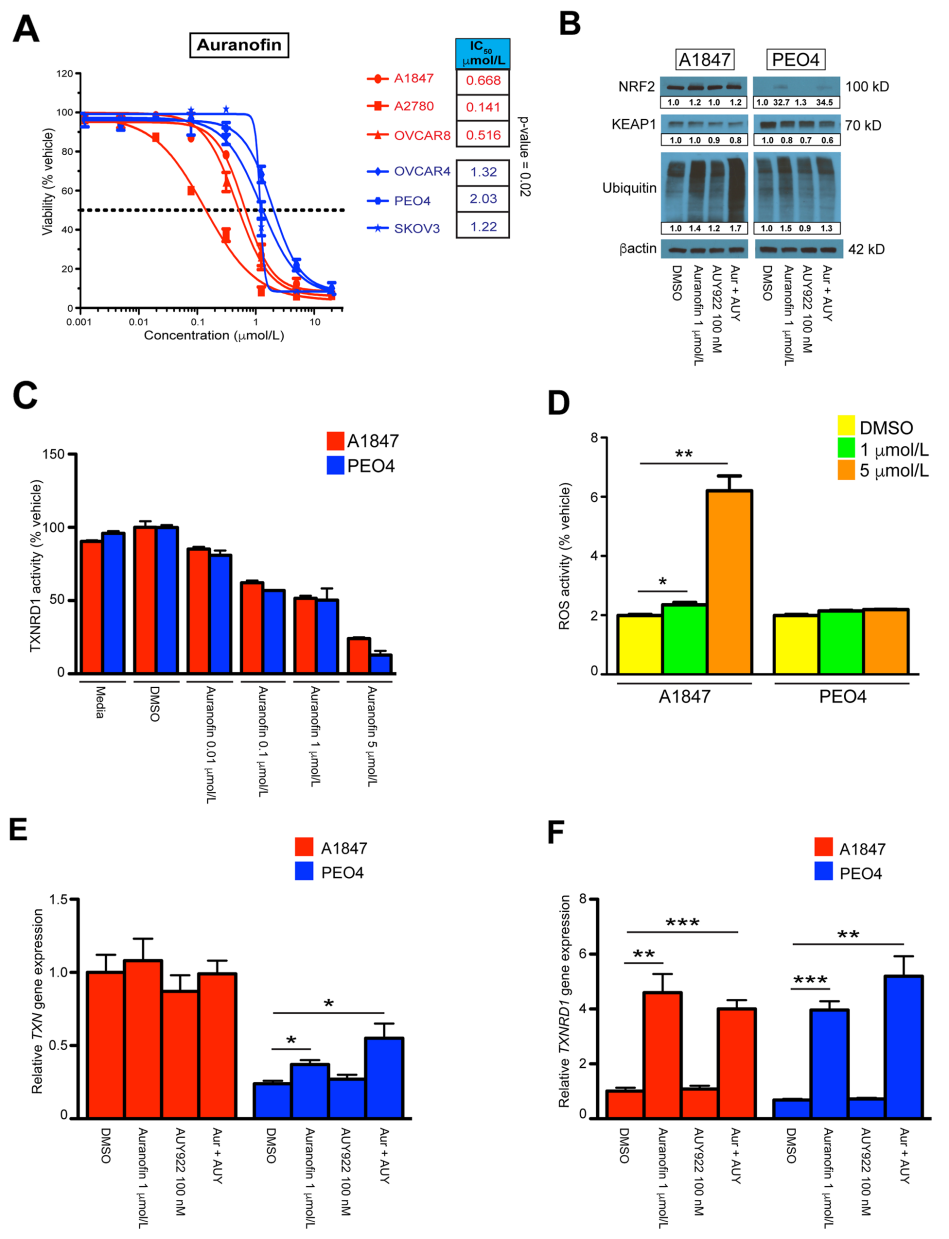
Single agent and combinatorial effects on reactive oxygen species homeostasis (**A**) IC_50_ values of auranofin treatment across A1847, A2780, OVCAR8 [sensitive] and OVCAR4, PEO4, SKOV3 [resistant] cell lines. The points represent average viability ± standard error of mean following 72 hours of drug treatment at the indicated concentrations. Curve-fit lines were generated using non-linear regression analysis in GraphPad Prism. (**B**) Protein expression of NRF2, KEAP1 and pan-ubiquitin 24 hours after incubation with indicated compounds, both alone and in combination (Aur + AUY). β-actin was used as a loading control and densitometric analysis of western blot data was performed using ImageJ software with band quantification relative to DMSO-treated samples. (**C**) Inhibition of TXNRD1 activity was measured by NADPH-dependent reduction of DTNB in A1847 and PEO4 cell lines 6 hours after incubation with the indicated compounds. (**D**) Measurement of total ROS levels was measured using DCF fluorescence in A1847 and PEO4 cell lines 6 hours after incubation with the indicated compounds. (**E, F**) Transcriptional expression of *TXN* and *TXRND1* in A1847 and PEO4 cell lines 6 hours after incubation with the indicated compounds, both alone and in combination (Aur + AUY). Data were quantified for the indicated fold-changes relative to vehicle treated cells and are presented as bar graphs showing the average fold-change ± standard error of the mean. ^*^ = p < 0.05, ^**^ = p < 0.01, ^***^ = p < 0.001.

We compared the auranofin IC_50_ dose response curves of our sensitive and resistant cell lines using a Student’s *t*-test and revealed a statistically significant difference between groups (Figure 2A). We next performed Western blot analyses of these cultures after incubation with the indicated concentrations (Figure 2B & Supplementary Figure 4A). The NRF2/KEAP1 pathway is a key regulator of cellular responses to oxidative stress in several ovarian cancer models [51, 52], and has been shown to be influenced by auranofin treatment [28, 53, 54]. We hypothesized that the effects of auranofin on ROS disruption are mediated, in part, through these proteins. We demonstrate that the sensitive A1847 cell line has strong basal expression of NRF2 while all other cell lines have diminished basal levels. Upon incubation with auranofin, either as a single agent or in combination with AUY922, the expression of NRF2 increased in all cell lines tested, indicating a common mechanism of action of auranofin. Conversely, protein expression of KEAP1 was similar between all cell lines at the basal level, though differences in KEAP1 inhibition were seen upon treatment with auranofin and/or AUY922 (Figure 2B & Supplementary Figure 4A). We also wanted to investigate the pan-ubiquitination status of proteins after incubation with auranofin due to its previous implication as an inhibitor of proteasome-associated deubiquitinases (DUBs) [30]. Our results showed that, although treatment with auranofin either alone or in combination with AUY922 increased total ubiquinated proteins across all lines tested, this phenomenon was markedly higher in the sensitive group, suggesting a role of DUB inhibition in the increased apoptotic effects of auranofin (Figure 2B & Supplementary Figure 4A).

Gold compounds such as auranofin have been shown to induce cell death through the deregulation of the thioredoxin reductase/thioredoxin (TXNRD1/TXN) redox system [55]. We therefore investigated the activity of our preferred drug combination on this system in EOC cell lines. TXNRD1 functions as a critical enzyme to maintain homeostasis of a reduced cellular milieu. Inhibition of TXNRD1 performance results in amplified levels of oxidized thioredoxin that further impairs the cellular response to oxidative stress. To determine if auranofin was inhibiting the enzymatic function of TXNRD1, we monitored TXNRD1 activity after 6 hours of auranofin exposure (Figure 2C & Supplementary Figure 4B). Interestingly, auranofin inhibits TXNRD1 activity at doses above 0.1 μmol/L in all cell lines tested, irrespective of its effects on viability (Supplementary Figure 4C). This finding demonstrates strong support that a principle mechanism of this gold compound is direct action on the TXNRD1/TXN system. To further delineate the influence auranofin has on the cellular response to impaired oxidative stress mechanisms, we performed RT-qPCR on sensitive A1847 and resistant PEO4 cell lines to examine transcriptional changes after treatment with auranofin and AUY922 (Figure 2E & 2F). We observed significant increases in the production of both *TXN* and *TXNRD1* transcripts in the resistant PEO4 line after 6 hours of auranofin as both a single agent and in combination, while the presence of AUY922 alone did not produce similar results. Both cell lines demonstrated significant increases in *TXNRD1* transcript production due to auranofin treatment, possibly due to a cellular compensatory mechanism for inhibited activity at the enzymatic level. Similar results were seen at 24 hours post-treatment, although by this time point even the sensitive A1847 line was increasing *TXN* transcriptional production due to auranofin exposure (Supplementary Figure 4D & 4E). We also wanted to measure differences in total ROS levels in the cells after exposure to these compounds. Although total ROS levels did not strictly correspond to cellular viability results between all cell lines (Supplementary Figure 5A), we did observe and increase in total ROS levels in the sensitive A1847 cell line 6 hours after treatment using a high concentration of auranofin (Figure 2D). This may indicate a higher sensitivity to auranofin on ROS homeostasis in A1847 as compared to PEO4. Finally, we investigated the effects of auranofin and AUY922 on mitochondrial function by monitoring disruptions in mitochondrial membrane potential. Changes in membrane potential lead to decoupling of the respiratory chain and is a key component of the early stages of programmed cell death. JC-1 is a dye that permeabilizes the mitochondrial membrane depending on its depolarization. The ratio of JC-1 aggregates-to-monomers is determined by the red/green fluorescence of JC-1 and is used as a general readout of mitochondrial membrane potential. However, we saw no changes in mitochondrial health of any cell line using this assay after treatment with auranofin, suggesting that the actions of auranofin are independent of mitochondrial stability (Supplementary Figure 5B).

### AUY922 mediated effects on sensitive and resistant cell models

**Figure 3:**
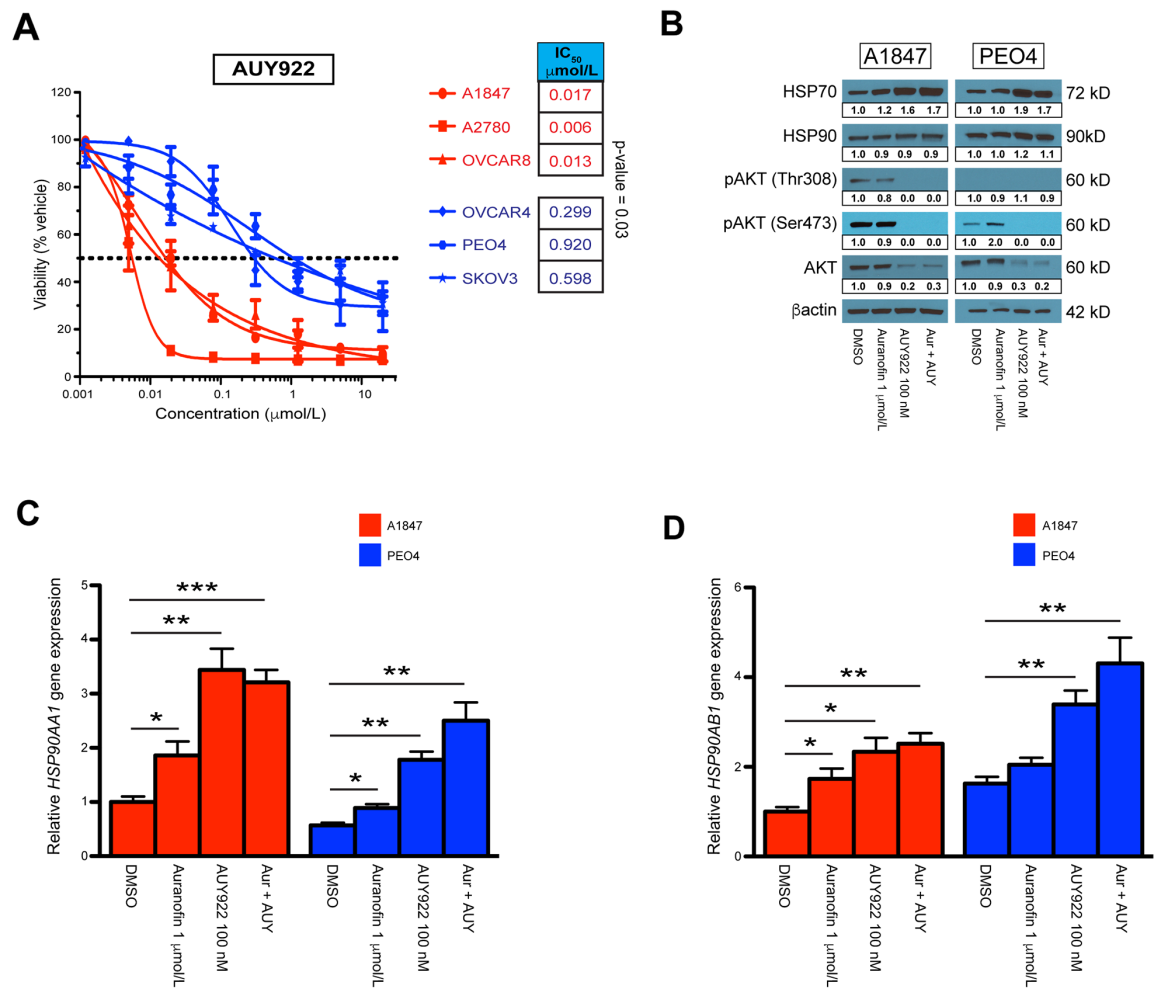
Single agent and combinatorial effects on the HSP90 pathway (**A**) IC_50_ values of AUY922 treatment across A1847, A2780, OVCAR8 [sensitive] and OVCAR4, PEO4, SKOV3 [resistant] cell lines. The points represent average viability ± standard error of mean following 72 hours of drug treatment at the indicated concentrations. Curve-fit lines were generated using non-linear regression analysis in GraphPad Prism. (**B**) Protein expression of HSP70, HSP90, pAKT-Thr^308^, pAKT-Ser^473^ and AKT 24 hours after incubation with indicated compounds, both alone and in combination (Aur + AUY). β-actin was used as a loading control and densitometric analysis of western blot data was performed using ImageJ software. (**C, D**) Transcriptional expression of the *HSP90AA1* and *HSP90AB1* subunits in A1847 and PEO4 cell lines 6 hours after incubation with the indicated compounds, both alone and in combination (Aur + AUY). Data were quantified for the indicated fold-changes relative to vehicle treated cells and are presented as bar graphs showing the average fold-change ± standard error of the mean. ^*^ = p < 0.05, ^**^ = p < 0.01, ^***^ = p < 0.001.

Similar to auranofin, we compared the AUY922 IC_50_ dose response curves of our cell lines (Figure 3A). Again, a Student’s *t*-test of the respective IC_50_’s demonstrated a significant p-value of 0.03 between groups. We next performed Western blot analysis 24 hours after incubation with indicated concentrations (Figure 3B, Supplementary Figure 6A & 6B). One of the hallmarks for effective HSP90 inhibition is an increase in the protein expression of HSP70 while HSP90 protein levels are maintained, as well as degradation of HSP90 client proteins [48, 56]. We observed this phenomenon across all cell lines using AUY922 either alone or in combination with auranofin. Additional verification that AUY922 efficacy is robust in all lines is the collective decrease in both phosphorylated AKT (at Thr^308^ and Ser^473^) and total AKT protein levels. We also wanted to observe transcriptional changes in HSP90 subunits after incubation with these compounds, therefore we performed RT-qPCR on sensitive A1847 and resistant PEO4 cell lines 6 hours after exposure (Figure 3C & 3D). We demonstrate significant increases in both the *HSP90AA1* and *HSP90AB1* subunits with either single agents, although AUY922 and the combination produced higher fold-changes then auranofin alone. Similar results were also seen at 24 hours post-incubation (Supplementary Figure 6C & 6D).

### Generation of predictive genetic signature

In order to evaluate the basal transcriptional activity of EOC cell lines that exhibit sensitivity versus resistance to the drug combination of auranofin and AUY922, RNA sequencing was performed to compare global expression profiles. We cultured the sensitive (A1847, A2780, OVCAR8) and resistant (OVCAR4, PEO4, SKOV3) lines, then extracted and purified total RNA. Paired end sequencing was performed and initial analyses were prepared using RSEM expected gene counts. The data were first filtered to remove non/low-expressed genes which resulted in a total of ∼14,000 genes that were examined for differential expression. Next, normalization factors were calculated to scale the library sizes followed by estimation of the tag wise negative binomial dispersion values. An exact test for differences in gene wise mean expression values was then implemented and provided both log fold change (logFC) and false discovery rate (FDR) values. We discovered 283 differentially expressed genes (FDR cutoff of <0.05) between the “sensitive” and “resistant” subgroups which allowed us to finalize the list by using the top 23 genes expressed in the resistant and sensitive cell lines as determined by logFC and FDR (Tables 1 & 2). We also compiled expected counts of sequenced isoforms from each signature and determined the total average counts per cell line as well as group average for each RefSeq ID (Supplementary Tables 2 & 3). In addition, we queried our RNAseq data for lncRNA expression and compiled three transcripts, including *HOTAIR*, that were highly expressed in sensitive cell lines while six lncRNAs were upregulated in the resistant group (Supplementary Table 4).

**Table 1:** List of genes representing the resistant genetic signature

Gene ID	Gene name	logFC	FDR
***CDH6***	cadherin 6, type 2, K-cadherin (fetal kidney)	9.9	1.77E-19
***IGFBP7***	insulin-like growth factor binding protein 7	12.2	1.82E-15
***MXRA5***	matrix-remodelling associated 5	13.7	1.10E-12
***ITGB6***	integrin, beta 6	10.6	1.07E-11
***WNT7A***	wingless-type MMTV integration site family, member 7A	11.7	6.96E-11
***TACSTD2***	tumor-associated calcium signal transducer 2	9.4	3.31E-09
***KRT19***	keratin 19, type I	9.3	3.78E-09
***ESR1***	estrogen receptor 1	13.0	6.46E-08
***MUC16***	mucin 16, cell surface associated	11.6	1.23E-07
***EHF***	ets homologous factor	9.5	2.28E-07
***S100A14***	S100 calcium binding protein A14	9.2	1.48E-06
***MAL2***	mal, T-cell differentiation protein 2	8.5	2.04E-08
***LAMA3***	laminin, alpha 3	7.7	1.94E-06
***ZBED2***	zinc finger, BED-type containing 2	9.1	6.53E-06
***LAD1***	ladinin 1	9.2	3.12E-05
***IRF6***	interferon regulatory factor 6	9.9	3.12E-05
***CFI***	complement factor I	9.2	3.35E-05
***CACNA2D3***	calcium channel, voltage-dependent, alpha 2/delta subunit 3	10.1	1.09E-04
***TSTD1***	thiosulfate sulfurtransferase (rhodanese)-like domain containing 1	10.5	1.78E-04
***COL5A1***	collagen, type V, alpha 1	8.8	7.63E-05
***MMP2***	matrix metallopeptidase 2	8.2	8.56E-05
***PDZK1IP1***	PDZK1 interacting protein 1	8.6	1.77E-04
***FAT2***	FAT atypical cadherin 2	8.0	1.89E-04

RNA sequencing analyses was condensed to Gene ID, Gene name, logFC (log2-fold change) and FDR (Benjamini-Hochberg adjusted p-value to account for multiple comparisons). The panel of 23 genes that exhibit the highest logFC and FDR in the three resistant cell lines as compared to the three sensitive lines were selected as the resistant signature.

**Table 2:** List of genes representing the sensitive genetic signature

Gene ID	Gene name	logFC	FDR
***IGF2BP1***	insulin like growth factor 2 mRNA binding protein 1	9.0	3.32E-06
***ITPRIPL1***	inositol 1,4,5-triphosphate receptor interacting protein-like 1	8.5	2.96E-05
***ELFN1***	extracellular leucine-rich repeat and fibronectin type III domain containing 1	9.7	3.12E-05
***CCND2***	cyclin D2	11.2	1.89E-04
***PLAC8***	placenta specific 8	6.0	3.71E-04
***ARHGAP28***	Rho GTPase activating protein 28	6.3	4.15E-04
***SERPINF1***	serpin peptidase inhibitor, clade F, member 1	7.9	8.03E-04
***NOS3***	nitric oxide synthase 3	5.1	1.49E-03
***TBX2***	T-box 2	7.4	1.68E-03
***TEX15***	testis expressed 15	7.7	2.00E-03
***RASGRP2***	RAS, guanyl releasing protein 2	7.4	2.24E-03
***BAHCC1***	BAH domain and coiled-coil containing 1	6.0	3.20E-03
***APC2***	adenomatosis polyposis coli 2	5.8	4.92E-03
***COL13A1***	collagen, Type XIII, Alpha 1	6.3	5.85E-03
***FREM2***	Fras1 related extracellular matrix protein 2	6.2	6.15E-03
***AGAP2***	ArfGAP with GTPase domain, ankyrin repeat and PH domain 2	6.7	6.37E-04
***MME***	membrane metallo-endopeptidase	5.6	9.19E-03
***ESPNL***	espin-like	6.1	9.31E-03
***DDR2***	discoidin domain receptor tyrosine kinase 2	6.1	0.01
***PDGFRB***	platelet-derived growth factor receptor beta	5.2	0.01
***SCARF1***	scavener receptor class F member 1	5.8	0.02
***COL3A1***	collagen type III alpha 1	7.1	0.03
***STAG3***	stromal antigen 3	6.2	0.04

RNA sequencing analyses was condensed to Gene ID, Gene name, logFC (log2-fold change) and FDR (Benjamini-Hochberg adjusted p-value to account for multiple comparisons). The panel of 23 genes that exhibit the highest logFC and FDR in the three sensitive cell lines as compared to the three resistant lines were selected as the sensitive signature.

### RT-qPCR validation of signature genes

Before moving on to high-density RT-qPCR analysis, we first wanted to validate the robustness of the dual signatures using conventional SYBR Green qPCR on a subset of genes. We designed primers and interrogated three genes from each list to determine if the *in silico* data was an accurate representation of the *in vitro* profiles. Primers targeting resistant genes (*CDH6*, *IGFBP7*, *WNT7A*) and sensitive genes (*ELFN1*, *ITPRIPL1*, *MME*) led to prominent discrepancies between cycle threshold (Ct) values from A1847 and PEO4 cell lines (Supplementary Figure 7A & 7B). In certain cases, no amplification curves were evident within 40 cycles, implying miniscule to null transcriptional activity of these genes. As predicted, A1847 mRNA transcripts related to the resistant signature demonstrated much higher Ct values as compared to PEO4 transcripts, with a minimal mean Ct variation of 10.9 ± 1.2 cycles. Similarly, PEO4 transcripts of the sensitive genes showed higher Ct values with a minimal mean of 8.6 ± 1.7 cycles. Notably, similar amplification curves were seen in the reference control gene *PPIA* in both lines, indicating that the disparate mRNA levels were not a global phenomenon but instead restricted to genes selected from RNA sequencing (Supplementary Figure 7A & 7B). We next wanted to determine if the transcriptional profile of A1847 and PEO4 cell lines persisted when grown *in vivo*. To that end, we extracted RNA from intraperitoneal tumors grown from these cell lines in NOD scid gamma (NSG) mice and investigated transcriptional levels of the above validation genes (Supplementary Figure 7C & 7D). Interestingly, the disparity of the resistant signature is dampened due to increased transcription in A1847 xenografts, as evident by lower Ct values resulting in a minimal mean difference of 3.9 ± 1.4 cycles. However, the sensitive validation genes displayed comparable differences in Ct between cell line xenografts with a minimal mean variation of 7.9 ± 1.3 cycles. Furthermore, *in vivo PPIA* expression remained comparable between tumor types (Supplementary Figure 7C & 7D).

**Figure 4:**
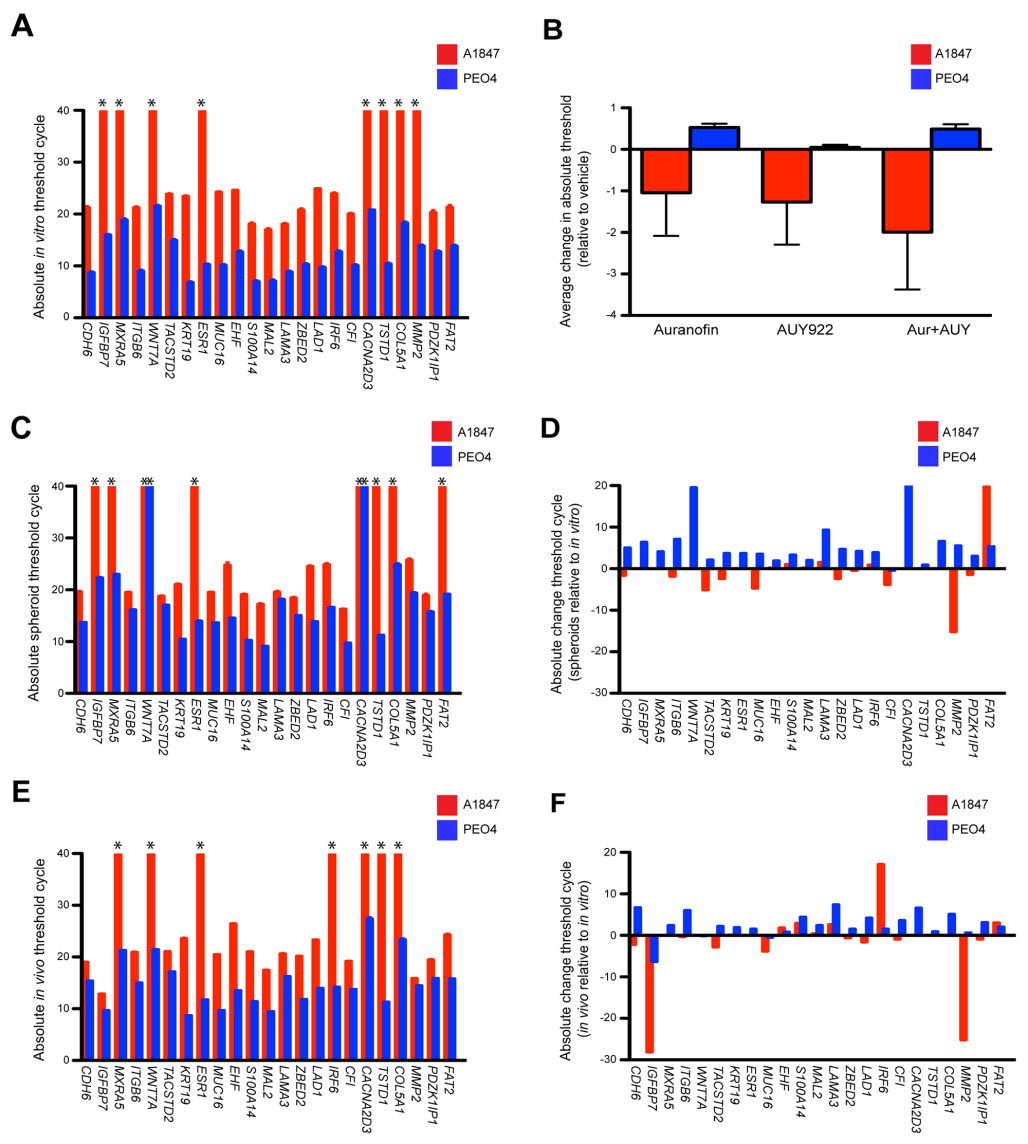
Expression of a 23-gene resistant signature using both *in vitro* and *in vivo* models (**A**) The 23-gene resistant profiles of A1847 and PEO4 cultured cells were evaluated utilizing TaqMan assays on the Biomark high-density qPCR system. Absolute cycle threshold values ± standard error of the mean from triplicate reactions of two independent experiments are graphed. Asterisks designate lack of amplification curves within 40 cycles. (**B**) Average variation of cycle thresholds for A1847 and PEO4 cultured cells treated with the indicated compounds, both alone and in combination (Aur + AUY). Data are displayed as bar graphs representing the difference of absolute threshold cycle ± standard error of the mean as compared to basal transcription from triplicate reactions of two independent experiments. Positive and negative columns signify higher and lower Ct values, respectively. (**C, E**) The 23-gene resistant profile was evaluated in A1847 and PEO4 spheroids and *in vivo* xenografts utilizing TaqMan assays on the Biomark high-density qPCR system. Absolute cycle threshold values ± standard error of the mean from triplicate reactions of two independent experiments are graphed. Asterisks designate lack of amplification curves within 40 cycles. (**D, F**) Absolute variation of cycle threshold for A1847 and PEO4 spheroids and *in vivo* xenografts compared to cultured cells. Data are displayed as bar graphs representing the difference between absolute threshold cycles using triplicate reactions of two independent experiments. Positive and negative columns signify higher and lower Ct values, respectively.

We then moved forward with high-density RT-qPCR studies by measuring mRNA expression of the 23 resistant signature genes in A1847 and PEO4 cultured cell lines using a 48x48 dynamic array on the Biomark™ HD system (Fluidigm microfluidic quantitative PCR platform). Using TaqMan® probes we determined basal Ct values of the resistant gene signature and demonstrate that they parallel the *in silico* data with a minimal mean variation of 15.9 (Figure 4A). We also used the Biomark assay to analyze gene signature fluctuations after 6 hours incubation with auranofin and AUY99, both as single agent and in combination (Supplementary Figure 8A, 8C & 8E). We observed moderate changes in Ct values 6 hours after drug treatment compared to basal levels with the greatest variations occurring in combination treated cells (Figure 4B & Supplementary Table 5). Similar to the SYBR Green results, the divergent gene expression of both basal and drug treated cultures is not reflected in the geometric mean of three reference controls *GUSB*, *PPIA* and *TBP* screened to confirm overall mRNA quality (Supplementary Figures 7E, 8B, 8D & 8F).

We next wanted to examine alterations in signature expression during the evolution towards a 3D tumor. To that end we forced the A1847 and PEO4 cultured cells into spheroid formation by culturing them in agarose-coated wells then extracted mRNA and compared Ct values for the resistant signature and reference controls from these spheroids (Figure 4C & Supplementary Figure 7F). Similar results were seen using mRNA from *in vivo* xenografts grown in mice (Figure 4E & Supplementary Figure 7G). Notable exceptions of genes displaying disparate Ct values as compared to the *in vitro* data are decreased expression of *WNT7A* and *CACNA2D3* in PEO4 spheroids as well as decreased expression of *FAT2* paired with increased expression of *MMP2* in A1847 spheroids (Figure 4D). Significant changes for *in vivo* xenografts as compared to *in vitro* cultured cells are an increase in *IGFBP7* and *MMP2* expression alongside a decrease in *IRF6* within the A1847 tumors (Figure 4F). Altogether, these results imply that the genetic machinery responsible for robust expression inherent to a given cell line may not be remarkably affected during the transformation from 2D to 3D models.

### Investigation of publicly available datasets for prevalence of genetic signatures

In order to determine whether EOC clinical samples are best represented by the resistant or sensitive signatures, we sought out the prevalence of these genes in publicly available databases. To substantiate the results produced from cell lines grown in the laboratory to those found in online resources, we first utilized the Cellminer database that queries molecular datasets focused on NCI-60 cell lines [57, 58]. Using this portal, we were able to extract average z-scores for our genes of interest from three of the cell lines used in our studies (OVCAR8, OVCAR4 & SKOV3) that reflected similar expression trends for both signatures (Supplementary Figure 9A & 9B). OVCAR8 displayed an average z-score of -0.5 for resistant genes and +0.6 for sensitive. Alternatively, OVCAR4 and SKOV3 show a +1.4 and +1.0 for resistant genes and -0.5 and -0.7 for sensitive, respectively. These results validate both the integrity of our cell lines as well as the robustness of the *in silico* data.

Recent interest in targeting the PRKCI pathway using auranofin as a treatment for ovarian cancer patients led us to investigate expression of this molecule in cell line models and to see if any correlation exists with our viability data. Using Cellminer, we determined the combined NCI-60 ovarian lines exhibited both the highest average z-score and protein RPLA levels relative to the other eight cancer types (Supplementary Figure 10A & 10B). We then performed copy number analysis on our sensitive and resistant cell line models that showed a modest trend toward increased *PRKCI* (the gene encoding protein kinase C iota) copy number in our resistant group (OVCAR4, PEO4 and SKOV3), though this difference was not significant (Supplementary Figure 10C). Finally, we analyzed if the log gene counts of our RNAseq data correlated with auranofin IC_50_ values across ten of our ovarian lines (Supplementary Figure 10D). The log gene count was elevated across all lines tested; further indicating that high expression of *PRKCI* is a common feature in ovarian cancer signaling. Although there is a significant Pearson correlation (0.66, p-value=0.036), this predictive value may be limited by the small dynamic range and high expression across all samples. Therefore, further investigation into the synergistic effects of PRKCI inhibition is warranted, but overall these data suggest that PRKCI is an attractive molecular target with enhanced specificity to ovarian cancer signaling.

**Figure 5:**
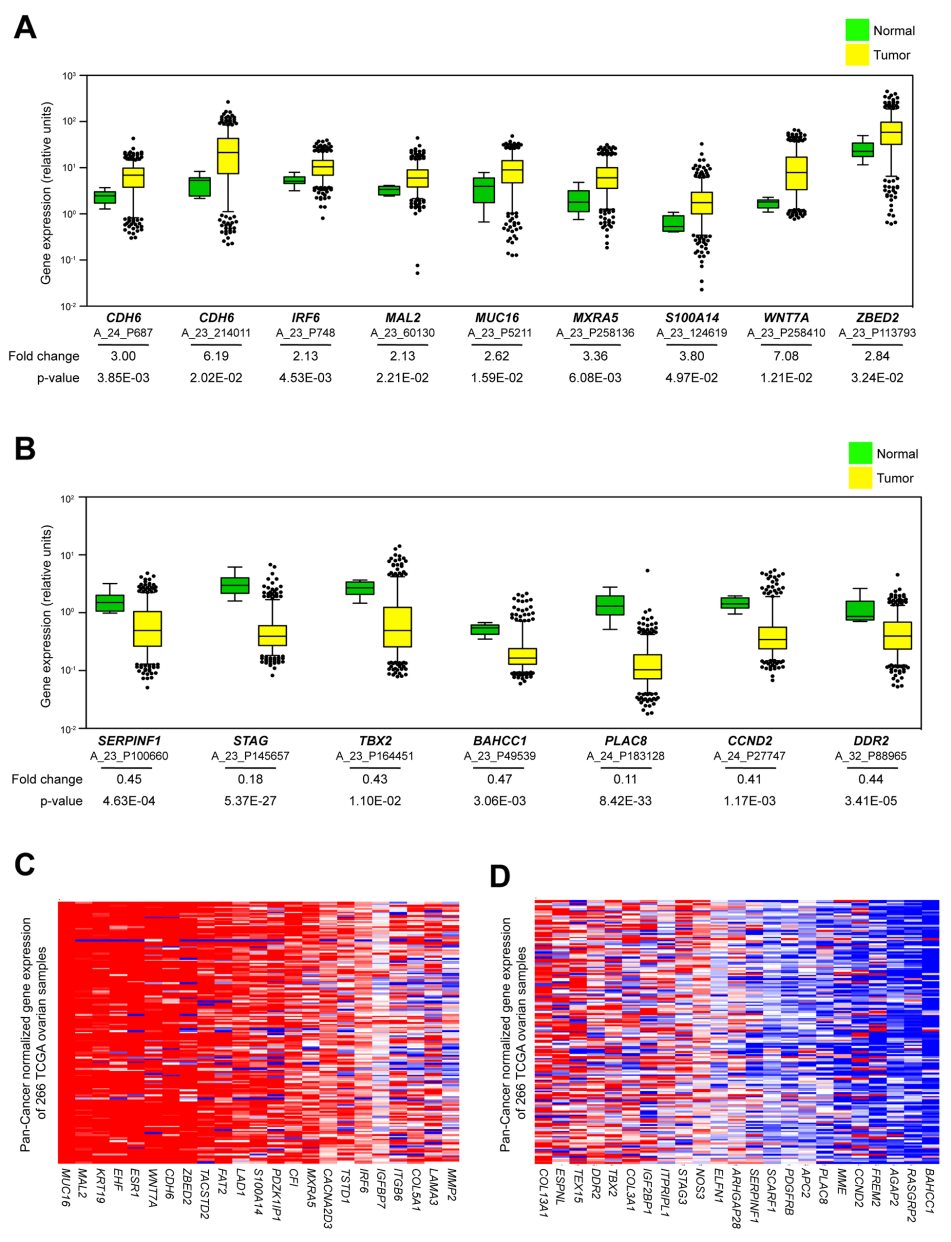
Investigation of publicly available datasets for prevalence of genetic signatures (**A**) Average expression values of Agilent probes related to an increase in the resistant signature from 518 TCGA tumors relative to 8 fallopian control samples. Data are displayed as box plots representing 8 genes (derived from 9 unique probes) that showed average increase of expression in tumors (yellow) greater than 2-fold increase over fallopian (green). (**B**) Average expression values of Agilent probes related to a decrease in the sensitive signature from 518 TCGA tumors relative to 8 fallopian control samples. Data are displayed as box plots representing 7 genes (derived from 7 unique probes) that showed average decrease of expression in tumors (yellow) greater than 2-fold increase over fallopian (green). The whiskers of each box plot represent expression values at 5^th^ and 95^th^ percentiles. Average fold change expression and p-values are indicated for each probe. (**C, D**) Heat map data of mRNA expression related to resistant and sensitive signatures from 266 TCGA serous ovarian patient samples sequenced using Illumina technology. All samples are mean-normalized per gene to 19 tumor types using Pan-Cancer analysis. Red and blue indicate increased and decreased mRNA expression, respectively.

To investigate if genes from our signatures are endemic in clinical EOC expression studies, we mapped TCGA Agilent probe IDs to our HGNC genes of interest. We then extracted gene expression data from 518 TCGA tumor samples and 8 normal fallopian tissues. Eight of the 23 genes in our resistant signature displayed average expression in tumor samples greater than 2-fold normal controls (*CDH6, IRF6, MXRA5, WNT7A, MUC16, MAL2, ZBED2* and *S100A14*) (Figure 5A & Supplementary Table 6). Alternatively, the sensitive signature showed only a single gene with significantly increased expression in tumors (*COL3A1*) (Supplementary Figure 9C & Supplementary Table 6), implying that these genes are less likely to be detected in large tumor panels compared to our selections based on resistance. Likewise, only a single gene from the resistant panel had higher expression in fallopian tissue compared to tumors (*IGFBP7*) (Supplementary Figure 9D, Supplementary Table 6), while 7 genes from the sensitive panel were shown to be expressed at significantly greater values in normal versus control tissue (*PLAC8*, *STAG3*, *DDR2*, *SERPINF1*, *BAHCC1*, *CCND2*, *TBX2*) (Figure 5B & Supplementary Table 6). This provides evidence that genes affiliated with resistance to our drug combination are more prevalent in tumor biology compared to those linked to sensitivity. Next, we determined the percentage of tumors that expressed individual Agilent probes greater than 2-fold of fallopian tissue (Supplementary Figure 9E & 9F). Again, expression of resistance genes was more common in the patients’ tumors (6 of the genes upregulated in > 50% of all tumor samples and 9 genes upregulated in < 20%) compared to those from the sensitive panel (2 of the genes upregulated in > 50% of all tumor samples with 18 genes upregulated in < 20%).

Finally, we wanted to investigate the relationship between our cell line signatures and the Pan-Cancer datasets that compile genomic information from TCGA studies across a multitude of cancer types [59, 60]. Assembling this mean-normalized RNA sequencing data provides the opportunity to evaluate genes of interest across a spectrum of cancer cohorts and is possible using the UCSC Cancer Genomics Browser (https://genome-cancer-ucsc.edu) [61]. Again, genes pertaining to our resistant signature appear much more likely to be detected within the 266 curated serous ovarian TCGA tumors than those from the sensitive list as compared to the sensitive signature (Figure 5C & 5D). Altogether, these studies provide justification that the cell lines displaying inherent resistance to the combination treatment of auranofin and AUY922 are superior models than the sensitive group for recapitulating the transcriptional profile of clinical EOC samples.

## DISCUSSION

Our motivation for this study was to determine if we could garner reliable information from 384-well high-throughput drug screening all the way through to existing clinical databases. The therapeutic potential provided would be feasible novel treatment options. We focused on the development of high-throughput viability screens of EOC cell lines paired alongside RNA sequencing technology to provide linkage between global gene expression and specific drug activity. To that end, we performed large-scale drug screening experiments with multiple ovarian cancer lines tested against a panel of clinically relevant compounds. Although we saw considerable potency from auranofin or AUY922 alone against several EOC cell lines, we focused on combining due to the inherent resistance displayed by other EOC lines, similar to what is seen among clinical responses using current chemotherapeutic protocols. We then investigated pathways downstream of these compounds and created multi-gene signatures linked to sensitivity or resistance. The availability of publically available genomic data through collections such as the TCGA allowed us to visualize the proclivity of our resistance signature within the patient population. Taken together, this study provides a platform to logically develop personalized treatments for patients with ovarian cancer.

By using a broad screening panel and correlating the response to genomic signatures, it may be possible to circumvent the diverse genetic complexity preventing successful treatment of late-stage EOC. Aberrant p53 and DNA repair functions in HGS tumors result in widespread genomic variance [2]. Relying solely on tissue of origin to determine treatment protocols is imprecise, instead the focus needs to shift to specific vulnerabilities inherent to individual tumors. Other tumor types, such as pancreatic ductal adenocarcinoma, demonstrate marked heterogeneity and have also proven difficult to treat successfully [62]. Even well characterized tumor types with dependable molecular biomarkers can be stratified into a spectrum of classifications previously unavailable using histologic or single-gene criteria. Therefore, the historical method of elucidating communal drivers intrinsic to certain tumor types to gauge effective therapeutic response may be better utilized by cataloging the efficacy of all available compounds against families of gene signatures. New and repurposed drug combinations could then be best matched to individual patients. The correlation between *in vitro* or clinical sequencing information to sensitivity data for the vast collection of FDA-cleared drugs can provide a system for therapeutic discovery.

Although randomized clinical trials have led to substantial advancement in understanding numerous disease processes, they have also resulted in needless treatment for patients who receive no benefit, which in turn leads to statistical insignificance of potentially effective compounds. In certain instances, targeted therapies have demonstrated improved response rates due to increased activity in subsets of tumors. The tyrosine kinase inhibitor imatinib mesylate (Gleevec®), for the treatment of Philadelphia chromosome-positive chronic myelogenous leukemia and KIT-positive gastrointestinal stromal tumors [63, 64], as well as the monoclonal antibody trastuzumab (Herceptin®) directed against HER2-positive breast cancer and gastroespohageal cancer [65], are two notable examples of targeted therapies with high activity in subsets of tumors. However, acquired resistance to single agent targeted therapy is commonplace and poses a substantial challenge to the management of aggressive cancers. Additionally, the cost and time commitment related to developing new therapies is prohibitive [66]. One potential explanation for the disparity between positive results in preclinical studies and negative results during human trials is a reliance on “disease-specific” cell culture models. Historically, a subset of cellular models has been utilized to recapitulate a wide spectrum of patient tumors resulting in unreliable activity during trials. As our studies show, a broad panel of mechanistic assays in ovarian cell lines demonstrated little commonality for signaling markers between the cell lines of the sensitive and resistant groups. Realistically, *in vitro* genomic alterations occur due to frequent passaging, variance in culturing techniques and/or extended cryopreservation. Therefore, performing preclinical studies using 2D cells that once demonstrated histological similarities to a given tumor type is an outdated and inefficient use of resources. Though dominant pathways may continue to drive proliferation among particular tumor cell line models, a more widespread molecular profile is warranted to determine the extent of genetic evolution and its usefulness as a clinical surrogate [67]. Researchers may find it best to first determine expression profiles from a tumor of interest and then screen for relevant cell lines that exhibit comparable signatures, even if that results in the use of atypical cell line models for the tumor type being evaluated. In our studies, the gene expression signatures from our resistant cell lines were more similar to clinical samples in the TCGA database than were the signatures of the sensitive group, thereby strengthening the importance of the resistant lines as the relevant tumor models.

It may prove more practical to investigate therapeutic intervention with existing FDA-approved compounds generated for other indications [68, 69]. However, one potential pitfall with a repurposing approach for existing compounds is achieving the clinically effective dosing needed in order for them to be used as chemotherapeutic agents. For example, although auranofin is helpful in reducing inflammation for arthritic patients, it has poor bioavailability when taken orally, with only 15-20% detected in plasma after dosing [25]. Two recent trials assessing auranofin as a monotherapy concluded with mixed results, possibly reflecting inefficient pharmacokinetics [36, 70]. Our recent studies of Ewing sarcoma further suggested that gold levels obtained via oral delivery of auranofin might be insufficient to achieve optimal anti-cancer effects [69]. Therefore, reformulation of the active gold component using intravenous or intraperitoneal delivery could potentially enhance the anti-tumoral activity of auranofin in ovarian cancer patients through both spatial targeting of the tumor and avoidance of the myriad of physiological factors affecting drug absorption via the gastrointestinal tract. Additionally, evaluating synergistic effects between repurposed classes of drugs may prove useful in circumventing resistant phenotypes through complementary blockade of discrete signaling pathways [71, 72]. Whereas auranofin as a single-agent has shown limited usefulness, multiple independent studies are investigating its benefit as a cooperative agent [28, 38–42, 73]. It is possible that the value of auranofin lies in contributing specific insults alongside other classes of chemotherapeutics. Heightened levels of ROS paired with inhibition of the proteasome ubiquitin system could overstress an adaptive tumor cell towards apoptosis. In our study, the use of auranofin in combination with an HSP90 inhibitor increased the effectiveness of the drug at lower concentrations, which could be beneficial to overcoming the clinical pitfalls seen with patient toxicities.

In order to make truly informed treatment decisions, a broader clinical evaluation of tumoral heterogeneity beyond single-pathway analysis is warranted. Cataloging genetic signatures could prove to be a sensible method for stratifying EOC patients into relevant targeted clinical trials [3, 74]. Microfluidic chips, RNAseq and digital multiplexed gene expression analysis have all demonstrated robustness and versatility using nucleic acid screening technologies [19, 75–77]. Global expression within individual tumors can now be generated using instrumentation available in clinical laboratories. Although the amount of raw sequencing data produced is abundant, it can be packaged into practical information using available bioinformatical toolboxes. Collective portals containing metadata of *in vitro* and *in vivo* metadata such as genetic signatures, isoform specificity and drug sensitivity information can be utilized by researchers to seek out pattern recognition across a milieu of cellular and tumor types, a process unfeasible using conventional detection methods. Resources such as the Pan-Cancer prognostic signatures utilized in this study will allow access to ∼11,000 human tumors encompassing 33 malignances from the TCGA data sets [78]. Additional inclusion of alternate molecular information, such as epigenomic and proteomic data, will lead to greater understanding of both tumor biology and pharmacogenomic influences. Burgeoning fields of study such as lncRNA activity allow for ample opportunity to detect patterns of expression in large, publically accessible datasets. lncRNA is a broad term denoting non-coding RNA greater than 200 nucleotides in length linked to various cancer types due to tissue-specific expression of 28,000 distinct transcripts mediating gene regulation and chromatin modification [79]. *HOTAIR*, an lncRNA originating in the *HOXC* cluster targets PRC2 complexes across chromosomes in the *HOXD* locus, has been linked to tumor progression in ovarian cancer. Recent studies involving both meta-analyses and gene signature construction are further gleaning the importance of lncRNAs, including *HOTAIR,* as biomarkers in this disease [80–82].

In conclusion, this study provides a platform to guide therapy based on widespread genomic signatures instead of single pathway inhibition. This is a promising approach to help tackle the treatment of ovarian cancer that lacks the common driver mutations necessary for targeted therapy. Instead, selection of drug combinations directed towards multiple cellular pathways associated with ovarian cancer progression may provide a platform for preselection based on genetic signatures.

## MATERIALS AND METHODS

### Compounds

Auranofin, AUY922 and ganetespib were purchased from Selleckchem. Upon receipt, dimethyl sulfoxide (DMSO) was used to prepare 10 mM stock solutions for all compounds except auranofin, which was prepared at 5 mM concentration due to reduced solubility. Single-use aliquots of stock solution were stored at -80°C.

### Cell culture

All cell lines used in this study were obtained or derived at the Fox Chase Cancer Center (Philadelphia, PA). Details of the origin of the EOC cell lines (A1847, A2780, C30, CP70, OVCAR4, OVCAR5, OVCAR8, OVCAR10, PEO4 and SKOV3) have been previously reported [83, 84]. All cell lines were grown in RPMI 1640 media (Corning Cellgro) containing 2 mM L-glutamine and supplemented with 10% FBS (Gibco), 100 U/mL penicillin (Corning Cellgro), 100 μg/mL streptomycin (Corning Cellgro), and 7.5 μg/mL insulin (Gibco) and maintained at 37°C in a humidified atmosphere with 5% CO_2_. Spheroids were formed using the liquid-overlay method with agarose coated 96-well plates. Briefly, 96-well flat bottom plates were coated with 50 μL 1.5% agarose (Sigma Aldrich). Agarose was dried for 30 minutes then 3,000 cells (A1847 or PEO4) were plated in media to commence spheroid formation. Fresh media was overlaid after four days and spheroids were collected after seven total days growth.

### Drug screening and cell viability measurements

The six EOC cell lines were grown to 80% confluency, harvested and seeded into 96-well plates at concentrations of 2000 to 4000 cells per well in a total volume of 95 μL. Twenty-four hours after seeding, drug compounds were prepared using cell growth media and 5 μL of each were added to the seeded cells of the 96-well plates. A Microlab Nimbus 96 pipetting robot (Hamilton) was used to prepare serial dilutions and drug addition to the cell lines. The final drug solutions consisted of eight concentrations ranging from 20 to 0.0012 μmol/L (serial four-fold dilutions). Vehicle-only wells were included on each plate to serve as interplate normalization controls. Seventy-two hours following drug addition, CellTiter Blue reagent (Promega) was added directly to each well using a Matrix WellMate (Thermo Scientific). The plates were incubated at 37 °C for 150 minutes and the fluorescent signal was measured using an Infinite® M200 Pro microplate reader (Tecan). The ratio of fluorescent signal in drug treated wells to that of average fluorescent signal from vehicle treated wells on each plate multiplied by 100 was calculated to yield percent cell viability for each drug treated well. A minimum of two biological replicates was performed for each cell line. Data analysis calculated IC_50_ values using Prism 5 software (GraphPad). All data in the viability curves are reported as mean ± standard error of the mean (SEM).

### Drug combination studies

In order to determine synergistic effects of the two compounds, EOC cells were seeded into 96-well plates as described above. Twenty-four hours after seeding, serial dilutions of auranofin, AUY922 or both were freshly prepared in DMSO/media and added to the wells either as single agent or a combination. Assays were performed as biological duplicates using triplicate wells within each experiment. Cell viability following 72 hours of treatment was evaluated using CTB as described above and CalcuSyn (Biosoft) software was used based on the Chou-Talalay method [50, 85]. The CalcuSyn software generates combination index (CI) values that determine the effect of the drug combination in comparison to the single compounds. A CI of <0.3 indicates strong synergistic effect between the two compounds, whereas a CI of 0.3 - 0.8 and >0.8 indicates synergistic and non-synergistic effect, respectively.

### Apoptosis

Annexin V labeling was performed using the Guava Nexin assay kit (Millipore) that contains a premixed cocktail of phycoerythrin-conjugated Annexin V and a cell impermeant dye (7-AAD). Cells in log phase of growth were grown to subconfluency, detached and seeded into 6-well plates at a concentration of 1×10^5^ - 2×10^5^ cells per well. Cells were allowed to attach overnight then treated with or without compound at the indicated concentrations. After 48 hours, cells were diluted to 5×10^5^ cells/mL and incubated with Guava Nexin reagent for 30 minutes. The assay was performed three independent times with three technical replicates each. Results were analyzed using a Guava easyCyte HT instrument (Millipore) and expressed as fold change of gated cells that are positive for Annexin V staining compared to vehicle control.

### Western blot

Cell lysates were prepared in M-PER Mammalian Protein Extraction Reagent (Thermo Scientific) with Mini protease and Halt phosphatase inhibitor cocktails (Thermo Scientific) and protein concentration was determined using the BCA assay (Thermo Scientific). 25 μg of total cellular protein was separated on SDS/PAGE gels and transferred to nitrocellulose membrane that was blocked for 1 hour at room temperature in 5% nonfat milk. Primary antibodies were added overnight at 4°C with gentle shaking. The primary antibodies used were: anti-TP53 (Sigma), anti-CDKN1A (Cell Signaling), anti-NRF2 (Abcam), anti-KEAP1 (Abcam), anti-ubiquitin (Cell Signaling), anti-HSP70 (Enzo), anti-HSP90 (Cell Signaling), anti-pAKTThr308 (Cell Signaling), anti-pAKTSer473 (Cell Signaling) and anti-AKT (Cell Signaling). After incubation with the appropriate secondary antibody, signals were detected using ECL Western Blotting Substrate (Thermo Scientific). Equal protein loading in each lane was confirmed with β-actin antibody (Sigma). Densitometry analysis was performed using the freely available image-processing program ImageJ (NIH).

### Thioredoxin reductase assay

Cells in log phase of growth were grown to subconfluency, detached and seeded into 6-well plates at a concentration of 5×10^5^ cells per well. Cells were allowed to attach overnight then treated with or without compound at indicated concentrations for 6 hours. Cells were lysed and 40 μg of total protein was used to measure TrxR activity using the TrxR Reductase Assay Kit (Abcam) according to manufacturer protocol.

### ROS activity measurement

Cells in log phase of growth were grown to subconfluency, detached and seeded into 96-well plates at a concentration of 2.5x10^4^ cells per well. ROS activity was measured using the Cellular Reactive Oxygen Species Detection Assay Kit (Abcam) according to manufacturer protocol. Briefly, cells were allowed to attach overnight, washed then treated with or without compound at indicated concentrations and timepoints. Cells were then exposed to 2’,7’-dichlorodihydrofluorescin diacetate (DCFH-DA) for 40 minutes and fluorescence was measured at 485/535 nm on a Tecan microplate reader.

### Copy number calculation

Total DNA was isolated from cultured cells using the JetFlex Genomic DNA Purification Kit (Invitrogen). Cells were lysed and subjected to Proteinase K prior to ethanol precipitation of DNA. An input concentration of 5 ng/μL was processed with a TaqMan® Copy Number Assay (Applied Biosystems). Results from a real-time PCR reaction were imported into CopyCaller™ Software (Applied Biosystems, version 2.1) that performs a Δ ΔC_T_ analysis which determines the relative copy number of the PRKCI gene normalized to the known copy number of RNAaseP.

### Real-time RT-PCR

Total RNA was extracted from EOC cell lines and spheroids using Trizol (Invitrogen) in Phase Lock Gel Heavy tubes (5Prime) then transitioned to RNeasy (Qiagen) columns with subsequent DNase treatment. For *in vivo* xenograft studies, A1847 and PEO4 cells were implanted intraperitoneally into female NOD.Cg-*Prkdc*^*scid*^*Il2rg*^*tm1Wjl*^/SzJ mice (Jackson). Mice were housed in our approved University Animal Facility with 12-hour light cycles, food/water were provided ad libitum. Institutional approval was granted for all experiments via an Animal Care and Use Protocol. Solid tumors were collected and tissue homogenized in Trizol using a Bullet Blender (Next Advance) followed by RNA isolation as above. cDNA was created from pooled RNA of three technical replicates using SuperScript III (Invitrogen) and amplification was performed on a CFX96 Real-Time System (Bio-Rad) using Maxima SYBR Master Mix (Thermo Scientific). All amplification reactions were performed at least twice using three technical replicates each and melting curve analyses were performed to ensure amplification specificity. Relative mRNA levels for each gene were assessed following normalization to an internal reference control (RC) *PPIA*. Threshold cycle (Ct) values were determined using amplification curves then normalized to RC expression to calculate ΔCt for each sample as follows: ΔCt_gene of interest_ = Ct_gene of interest_ – Ct_RC_. The amount of mRNA in drug treated cells relative to vehicle treated cells was calculated as follows: 2^-ΔΔCt^, where ΔΔCt = ΔCt_drug treated gene of interest_ – ΔCt_vehicle treated gene of interest_. Due to large disparities in Ct values between A1847 and PEO4 in signature validation and high-density RT-qPCR studies, the customary 2^-ΔΔCt^ method for comparing relative mRNA expression was abandoned and absolute Ct values provided. Bio-Rad CFX Manager and GraphPad Prism software were used for statistical analyses and graphs. Primer sequences are described in Supplementary Methods.

### Generation of the dual genetic signatures

Total RNA was extracted as described above and prepared for paired end sequencing on a HiSeq 2500 using a stranded library prep kit (Illumina). Initial analyses were prepared using RSEM expected gene counts. First, data were filtered to remove non/low-expressed genes. This resulted in a total of ∼14,000 genes that were examined for differential expression between the grouped sensitive versus resistant cell lines. Next, normalization factors were calculated to scale the library sizes followed by estimation of tag wise negative binomial dispersion values. An exact test was implemented for differences in gene wise mean expression values between resistance and sensitivity phenotypes. Genes were then ranked according to both log fold change and multiple testing adjusted false discovery rate (FDR) q-values. Expected isoform counts of corresponding genes were matched to RefSeq IDs and tabulated for each cell line. lncRNA quantification was extracted from RNAseq data by RefSeq IDs using the statistical software R, then pair-wise comparison between sensitive and resistant cell line groups was conducted using the edgeR bioconductor package.

### High-density RT-PCR

The Fluidigm BioMark™ HD system was used to run 48x48 dynamic arrays to measure basal mRNA expression levels in EOC cell lines, spheroids and xenografts. Total RNA was extracted as described above and three biological replicates were pooled for analysis. 250 ng of mRNA were reverse transcribed and the resulting cDNA was pre-amplified using a multiplexed specific target amplification protocol with gene specific TaqMan assays (Applied Biosystems). The targeted cDNA was diluted 5-fold and used as input cDNA for qPCR arrays on the BioMark following manufacturer recommended protocols. Absolute Ct values were determined using amplification curves and equality of amplifiable mRNA was assessed by comparing geometric means of three internal reference controls; *GUSB, PPIA* and *TBP*. GraphPad Prism software was used for statistical analyses and graphs.

### *In silico* interrogation of publicly available databases

The CellMiner™ analysis tool (http://discover.nci.nih.gov/cellminer/analysis.do) was used to query NCI-60 cell line signatures. It provides average z-scores and RPLA protein levels from genes of interest for the available lines. Analysis of the TCGA microarray gene expression dataset was performed using the log_2_ transformed Agilent values for the genes of interest from 518 serous cystadenocarcinoma patients and 8 organ-specific healthy control samples (http://tcga-data.nci.nih.gov/tcga/tcgaHome2.jsp). Anti-log values were determined and mean expression values for all samples were calculated. The fold-change of average expression in the tumors relative to controls was constructed and a Student’s two-tailed *t*-test was performed. A fold-change of ≥ 2 with an associated p-value < 0.05 was set as a significant difference in expression between groups. The UCSC Cancer Genome Browser portal was used for investigation of direct RNA sequencing comparisons (https://genome-cancer.ucsc.edu/). It enables heat map construction using level 3 TCGA ovarian data from 266 samples that is mean normalized to 19 tumor types from the Pan-Cancer datasets.

## SUPPLEMENTARY MATERIALS FIGURES AND TABLES


