# Supplementary material for: Developing a genetic signature to predict drug response in ovarian cancer
Source: Oncotarget. 2017 Dec 26;9(19):14828–48. doi: 10.18632/oncotarget.23663 (PMC5871081; doi:10.18632/oncotarget.23663)
Supplement: Supplementary file 1 [file oncotarget-09-14828-s001.pdf]

Developing a genetic signature to predict drug response in ovarian cancer

SUPPLEMENTARY MATERIALS

JC-1

Cells in log phase of growth were grown to subconfluency, detached and seeded into 96-well plates at a concentration of 1×10^5 cells per well. Cells were allowed to attach overnight, washed then treated with or without compound at indicated concentrations and time points. Mitochondrial membrane potential was evaluated using the JC-1 Mitochondrial Membrane Potential Assay kit (Cayman Chemical) according to manufacturer protocol. Briefly, cells were exposed to JC-1 Staining Solution and incubated for 20 minutes at 37°C. The ratio of fluorescent intensity of J-aggregates to monomers was used as an indicator of mitochondrial membrane potential.

RT-qPCR primers

The following primers were used with SYBR® Green RT-PCR: Qiagen RT2 qPCR Primer Assay for Human TXN, PPH18986-200; Qiagen RT2 qPCR Primer Assay for Human TXNRD1, PPH02104F-200; Qiagen RT2 qPCR Primer Assay for Human HSP90AA1, PPH163391B-200; Qiagen RT2 qPCR Primer Assay for Human HSP90AB1, PPH01201C-200; IDT CDH6, GGAGGACAGGGAACAGTATTT, GAGCCGATATG CCCTCATTT; IDT WNT7A, CACTGGTGCTGCT ATGTCAA, AGTACCCTCCTCAGCAGAAA; IDT IGFBP7, GTACTTGAGCTGTGAGGTCATC, CACCCA GCCAGTTACTTCAT; IDT ELFN1, GCCCTG ACACACAACAGGAA, GGGTGTCCTGTCCCAAACC; IDT ITPRIPL1, GCTCGGACAAGCATGGTCTA, GCCTCTGCATCGTCAGTGTT; IDT MME, CTGCTGAGGGGTCACGATTTTA, AGGACCGAG AGGCTGATCTC. IDT PPIA, AGACAAGGTCC CAAAGAC, ACCACCCTGACACATAAA.

The following primers were used with TaqMan® RT-PCR: IGFBP7, Hs00266026_m1; KRT19, Hs01051611_gH; ESR1, Hs00174860_m1; MUC16, Hs01065189_m1; EHF, Hs00171917_m1; LAMA3, Hs00165042_m1; MMP2, Hs01548727_m1; CDH6, Hs01026780_m1; MXRA5, Hs01019147_m1; ITGB6, Hs00982345_m1; WNT7A, Hs00171699_m1; TACSTD2, Hs01922976_s1; S100A14, Hs04189107_g1; MAL2, Hs00294541_m1; ZBED2, Hs00976682_s1; IRF6, Hs01062178_m1; CACNA2D3, Hs01045015_m1; TSTD1, Hs00939899_g1; COL5A1, Hs00609133_m1; PDZK1IP1, Hs00173779_m1; FAT2, Hs01087225_m1; LAD1, Hs00194326_m1; CFI, Hs00989715_m1; GUSB, Hs00939627_m1; PPIA, Hs04194521_s1; TBP, Hs00427620_m1.

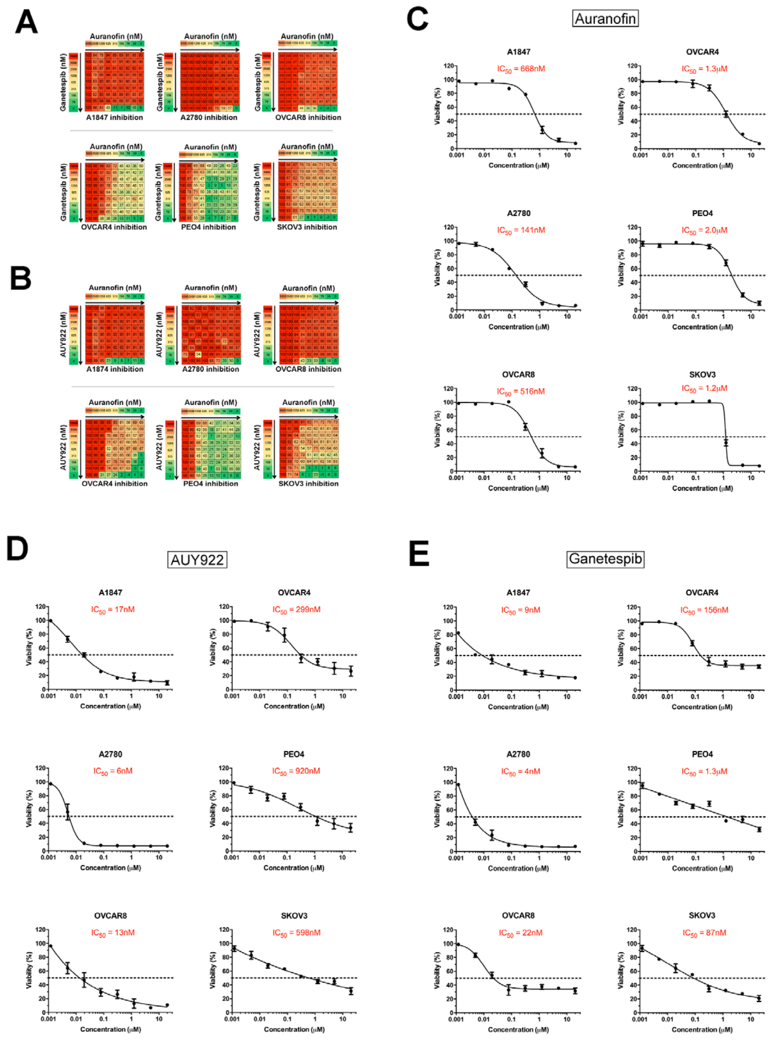

Supplementary Figure 1: Viability studies for auranofin and the HSP90 inhibitors AUY922 and ganetespib in sensitive and resistant cell lines. (A) Color scale for the percentage of viability inhibition is shown across 64 different drug combinations of auranofin and ganetespib for sensitive (upper) and resistant (lower) cell lines. **(B)** Color scale for the percentage of viability inhibition is shown across 64 different drug combinations of auranofin and AUY922 for sensitive (upper) and resistant (lower) cell lines. **(C)** IC_{50} values of auranofin treatment across six different EOC cell lines. The points represent average viability ± standard error of mean following 72 hours of drug treatment at the indicated concentrations. Curve-fit lines were generated using non-linear regression analysis in GraphPad Prism. **(D)** IC_{50} values of AUY922 treatment across six different EOC cell lines. The points represent average viability ± standard error of mean following 72 hours of drug treatment at the indicated concentrations. Curve-fit lines were generated using non-linear regression analysis in GraphPad Prism. **(E)** IC_{50} values of ganetespib treatment across six different EOC cell lines. The points represent average viability ± standard error of mean following 72 hours of drug treatment at the indicated concentrations. Curve-fit lines were generated using non-linear regression analysis in GraphPad Prism.

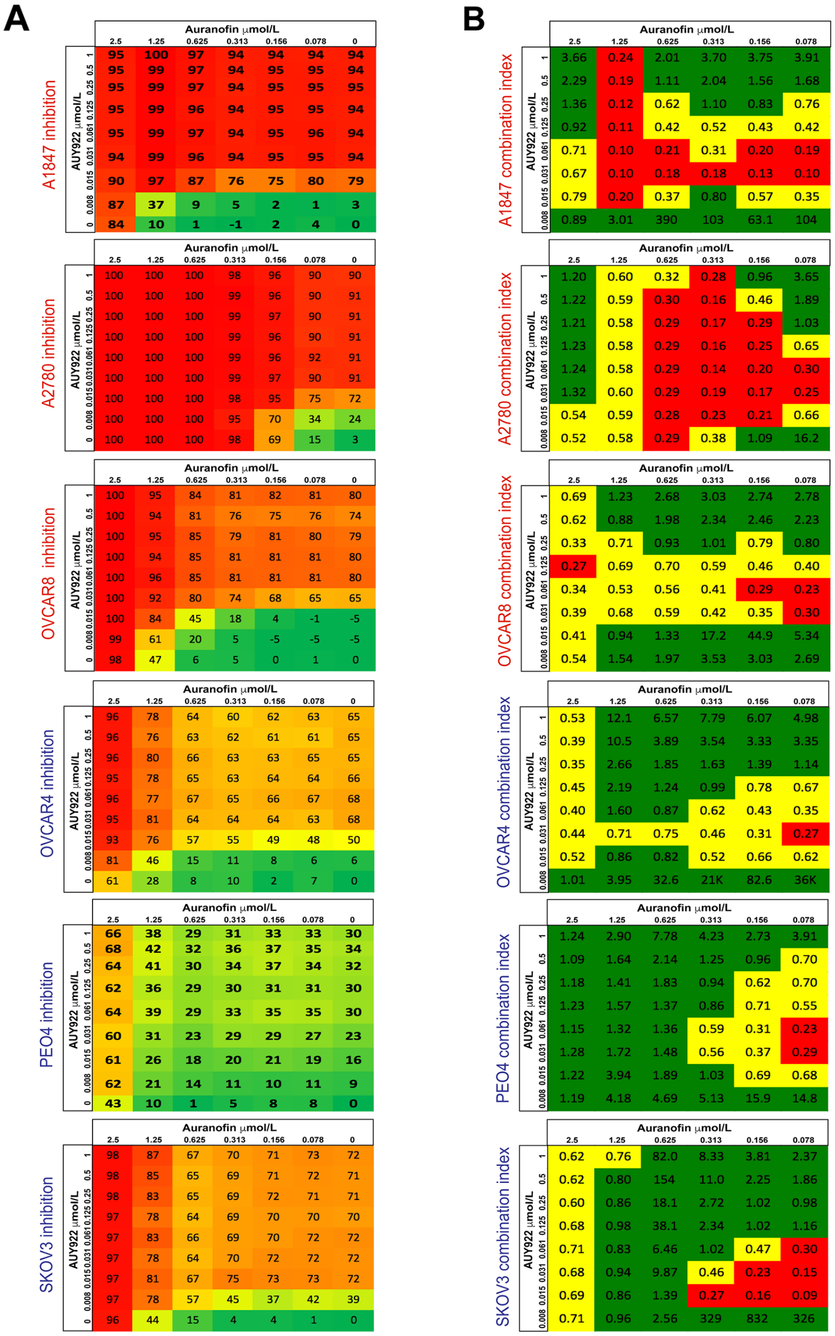

Supplementary Figure 2: Synergistic effects on of auranofin and AUY922 in sensitive and resistant cell lines. (A) Color scale for percentage of viability inhibition is shown across 48 different drug combinations of auranofin and AUY922 for sensitive (A1847, A2780, OVCAR8) and resistant (OVCAR4, PEO4, SKOV3) cell lines. **(B)** The dose response data was used to calculate the Combination index (CI) values for auranofin and AUY922 combination treatment for sensitive (A1847, A2780, OVCAR8) and resistant (OVCAR4, PEO4, SKOV3) cell lines. Shown is the average calculated CI value ± standard error of the mean. CI value of >0.8 [green] indicates no synergy; CI = 0.3-0.8 [yellow] indicates synergistic effects; CI value of <0.3 [red] indicates strong synergistic effects.

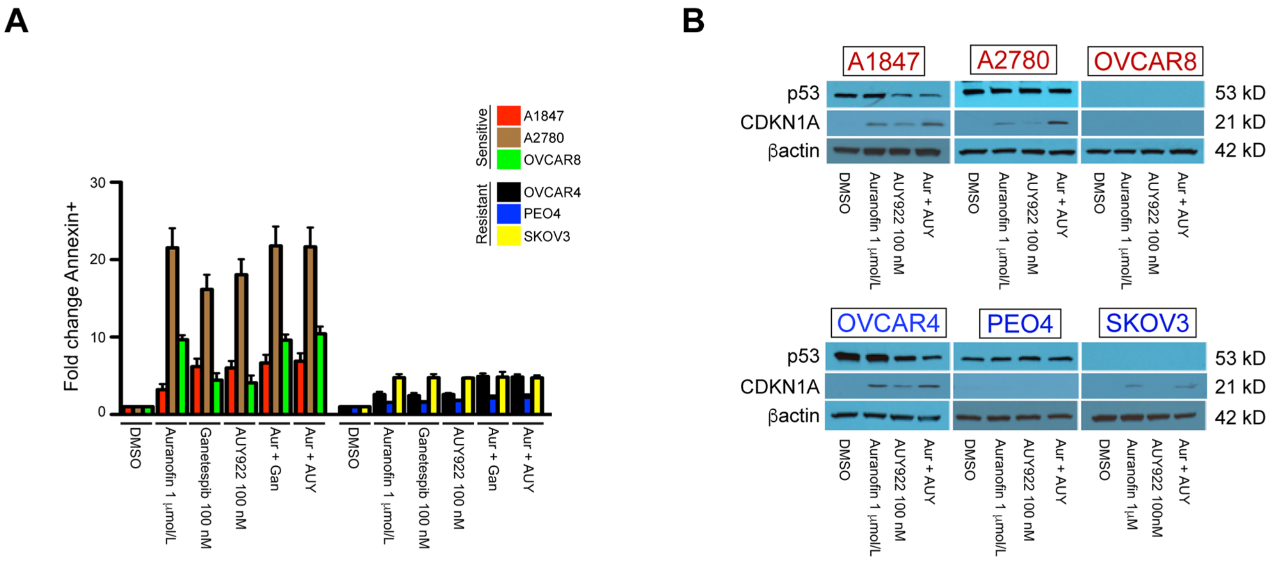

Supplementary Figure 3: Single agent and combinatorial effects of auranofin and AUY922 on apoptosis. **(A)** Fold-change of all sensitive and resistant cells positive for Annexin V staining 48 hours after incubation with indicated compounds, both alone and in combination (Aur + Gan and Aur + AUY). Data were quantified for the indicated fold-changes relative to vehicle treated cells and are presented as bar graphs showing the average fold-change ± standard error of the mean. **(B)** Protein expression of TP53 and CDKN1A 24 hours after incubation with indicated compounds, both alone and in combination (Aur + AUY). β-actin was used as a loading control.

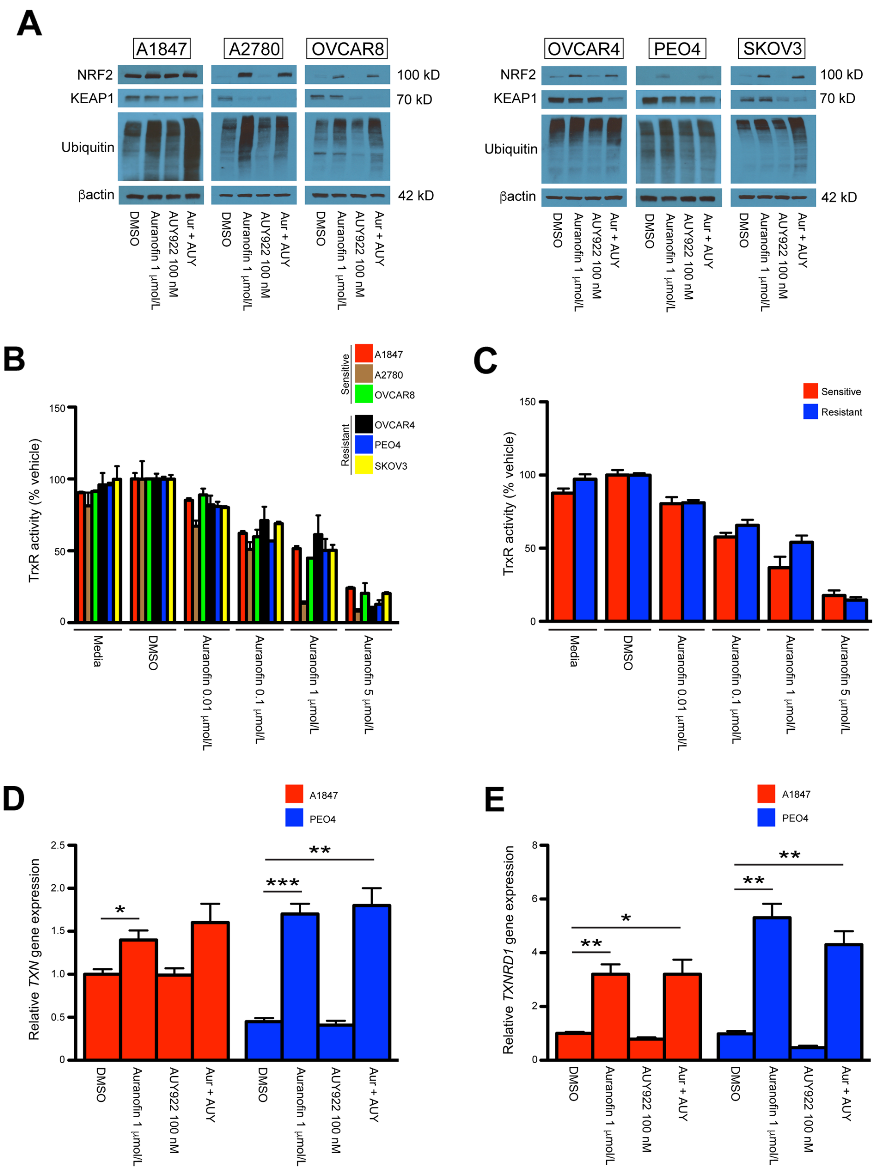

Supplementary Figure 4: Single agent and combinatorial effects of auranofin and AUY922 on reactive species homeostasis. **(A)** Protein expression of NRF2, KEAP1 and pan-ubiquitin 24 hours after incubation with indicated compounds, both alone and in combination (Aur + AUY). β-actin was used as a loading control. **(B)** Inhibition of TXNRD1 activity in sensitive (A1847, A2780, OVCAR8) and resistant (OVCAR4, PEO4, SKOV3) cell lines 6 hours after incubation with increasing concentrations of auranofin. **(C)** Collective inhibition of TXNRD1 activity in sensitive (A1847, A2780, OVCAR8) and resistant (OVCAR4, PEO4, SKOV3) cell lines 6 hours after incubation with increasing concentrations of auranofin. **(D, E)** Transcriptional expression of *TXN* and *TXNRD1* in A1847 and PEO4 cell lines 24 hours after incubation with the indicated compounds, both alone and in combination (Aur + AUY). Data were quantified for the indicated fold-changes relative to vehicle treated cells and are presented as bar graphs showing the average fold-change ± standard error of the mean. * = $p < 0.05$, ** = $p < 0.01$, *** = $p < 0.001$.

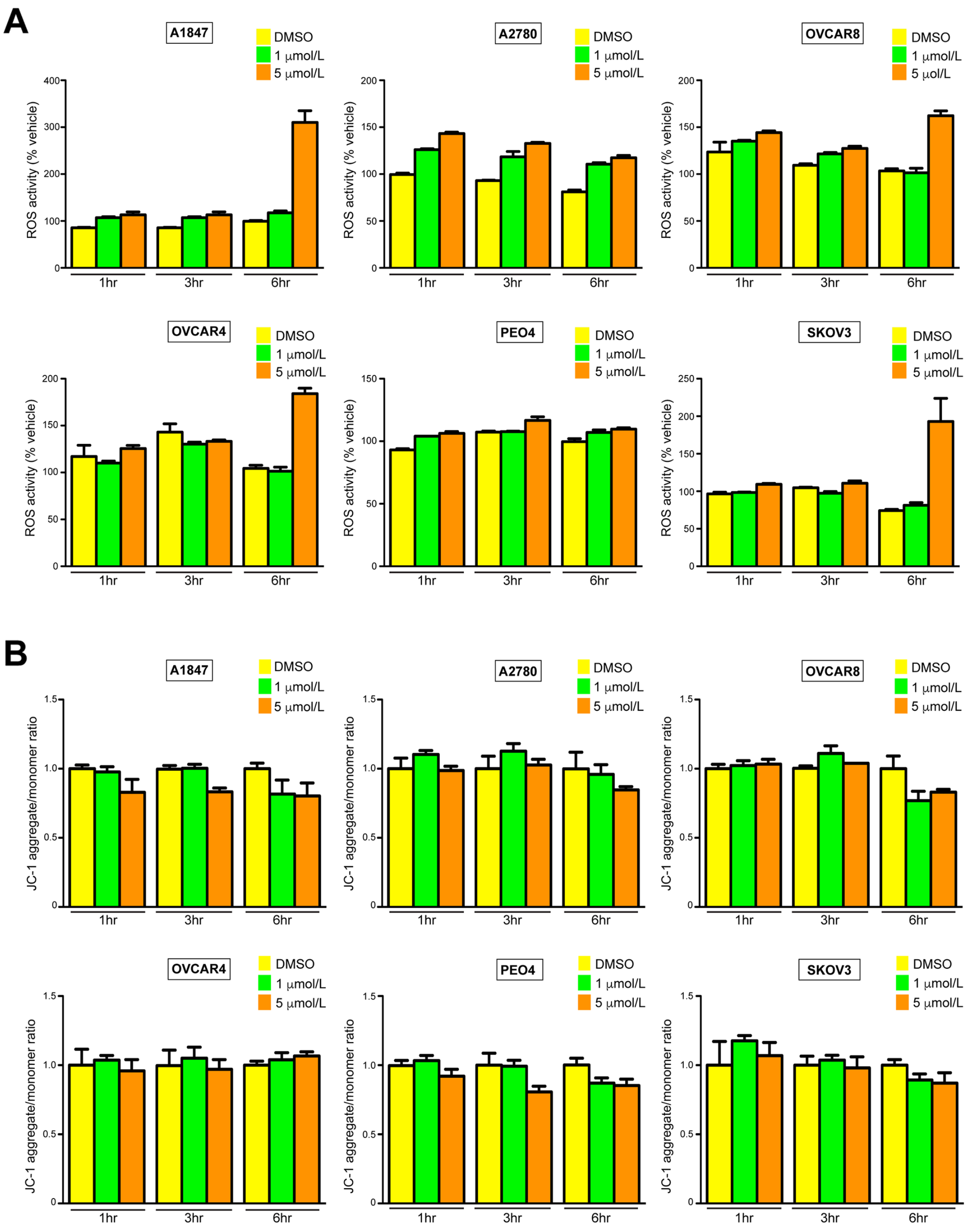

Supplementary Figure 5: Single agent effects of auranofin on reactive oxygen species homeostasis. **(A)** Measurement of ROS levels using DCF fluorescence after 6 hours auranofin treatment at the indicated concentrations for sensitive (A1847, A2780, OVCAR8) and resistant (OVCAR4, PEO4, SKOV3) cell lines. **(B)** Measurement of the JC-1 aggregate ratio after 6 hours auranofin treatment at the indicated concentrations for sensitive (A1847, A2780, OVCAR8) and resistant (OVCAR4, PEO4, SKOV3) cell lines. Data were quantified for the indicated fold-changes relative to vehicle treated cells and are presented as bar graphs showing the average fold-change ± standard error of the mean.

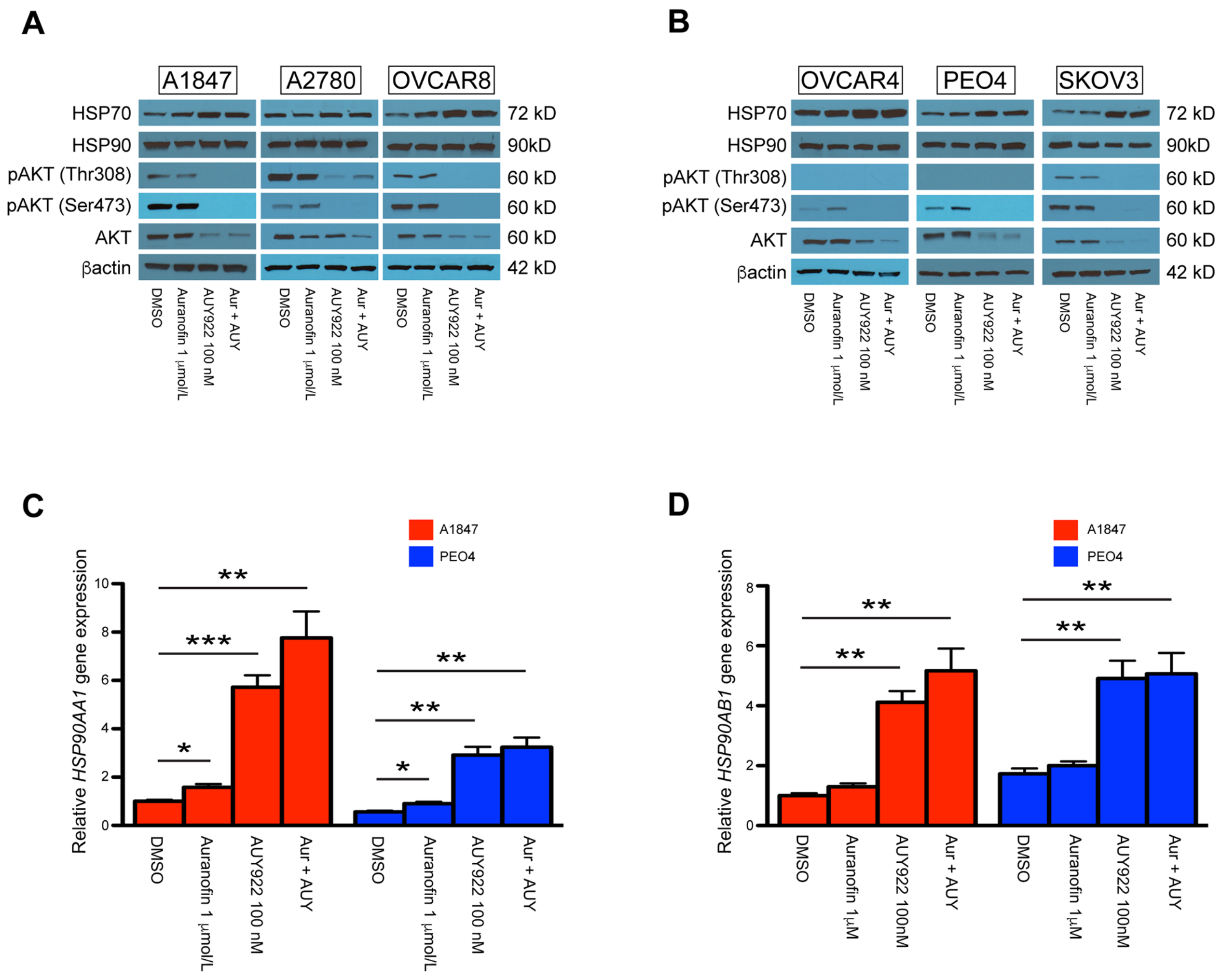

Supplementary Figure 6: Single agent and combinatorial effects on the HSP90 pathway. (A, B) Protein expression of HSP70, HSP90, pAKT-Thr308, pAKT-Ser473 and AKT for sensitive (A1847, A2780, OVCAR8) and resistant (OVCAR4, PEO4, SKOV3) cell lines 24 hours after incubation with indicated compounds, both alone and in combination (Aur + AUY). β-actin was used as a loading control. **(C, D)** Transcriptional expression of the *HSP90AA1* and *HSP90AB1* subunits in A1847 and PEO4 cell lines 24 hours after incubation with the indicated compounds, both alone and in combination (Aur + AUY). Data were quantified for the indicated fold-changes relative to vehicle treated cells and are presented as bar graphs showing the average fold-change ± standard error of the mean. $^{*} = p < 0.05$, $^{**} = p < 0.01$, $^{***} = p < 0.001$.

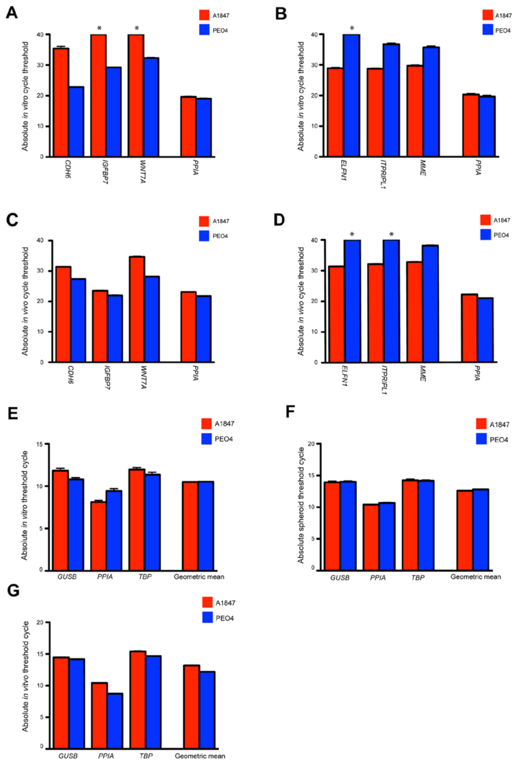

Supplementary Figure 7: Validation of genetic signature using real-time quantitative reverse transcription PCR. (A) Basal transcriptional expression of genes from the resistant genetic signature *CDH6*, *IGFBP7*, *WNT7A* and the reference control *PPIA* in A1847 and PEO4 cultured cells. Data are displayed as bar graphs representing the absolute threshold cycle ± standard error of the mean from triplicate reactions of two independent experiments. Asterisks designate lack of amplification curves within 40 cycles. **(B)** Basal transcriptional expression of genes from the sensitive genetic signature *ELFN1*, *ITPRIPL1*, *MME* and the reference control *PPIA* in A1847 and PEO4 cultured cells. Data are displayed as bar graphs representing the absolute threshold cycle ± standard error of the mean from triplicate reactions of two independent experiments. Asterisks designate lack of amplification curves within 40 cycles. **(C)** Basal transcriptional expression of genes from the resistant genetic signature *CDH6*, *IGFBP7*, *WNT7A* and the reference control *PPIA* in A1847 and PEO4 from *in vivo* xenografts. Data are displayed as bar graphs representing the absolute threshold cycle ± standard error of the mean from triplicate reactions of two independent experiments. **(D)** Basal transcriptional expression of genes from the sensitive genetic signature *ELFN1*, *ITPRIPL1*, *MME* and the reference control *PPIA* in A1847 and PEO4 from *in vivo* xenografts. Data are displayed as bar graphs representing the absolute threshold cycle ± standard error of the mean from triplicate reactions of two independent experiments. Asterisks designate lack of amplification curves within 40 cycles. **(E-G)** Three reference controls genes; *GUSB*, *PPIA* and *TBP* were evaluated along with their geometric mean using the Biomark high-density qPCR system utilizing TaqMan assays for the *in vitro*, spheroid formation and *in vivo* experiments, respectively. Average cycle threshold values ± standard error of the mean from triplicate reactions of two independent experiments are graphed.

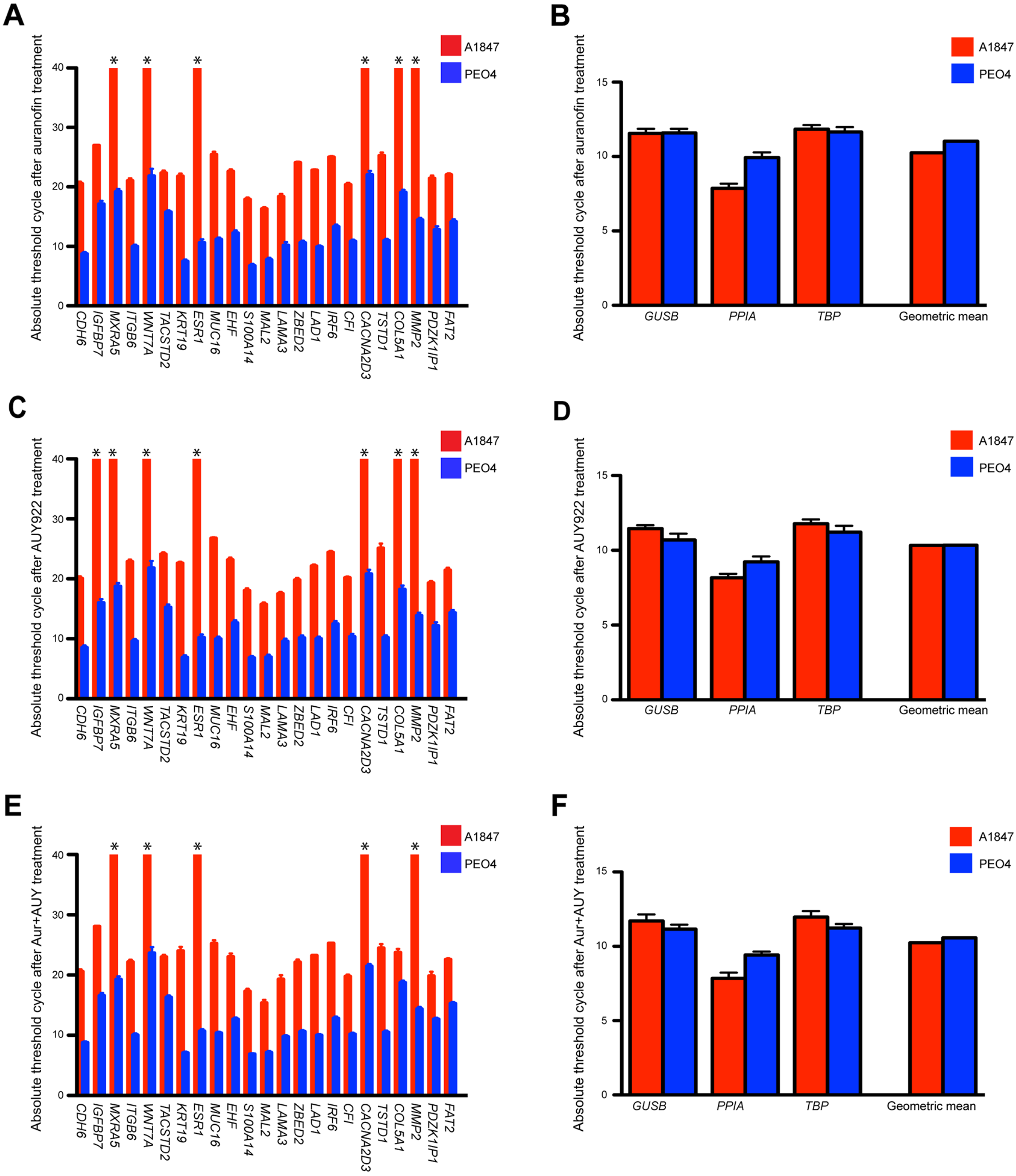

Supplementary Figure 8: Transcriptional variation of resistant signature after exposure to auranofin and AUY922. (A, B) Transcriptional expression of 23 genes from the resistant genetic signature as well as three reference control genes; *GUSB*, *PPIA* and *TBP* alongside their geometric mean in A1847 and PEO4 cultured cells after incubation with 1 μmol/L auranofin for six hours. **(C, D)** Transcriptional expression of 23 genes from the resistant genetic signature as well as three reference control genes; *GUSB*, *PPIA* and *TBP* alongside their geometric mean in A1847 and PEO4 cultured cells after incubation with 100 nM AUY922 for six hours. **(E, F)** Transcriptional expression of 23 genes from the resistant genetic signature as well as three reference control genes; *GUSB*, *PPIA* and *TBP* alongside their geometric mean in A1847 and PEO4 cultured cells after incubation with 1 μmol/L auranofin and 100 nM AUY922 for six hours. Data are displayed as bar graphs representing the absolute threshold cycle ± standard error of the mean from triplicate reactions of two independent experiments. Asterisks designate lack of amplification curves within 40 cycles.

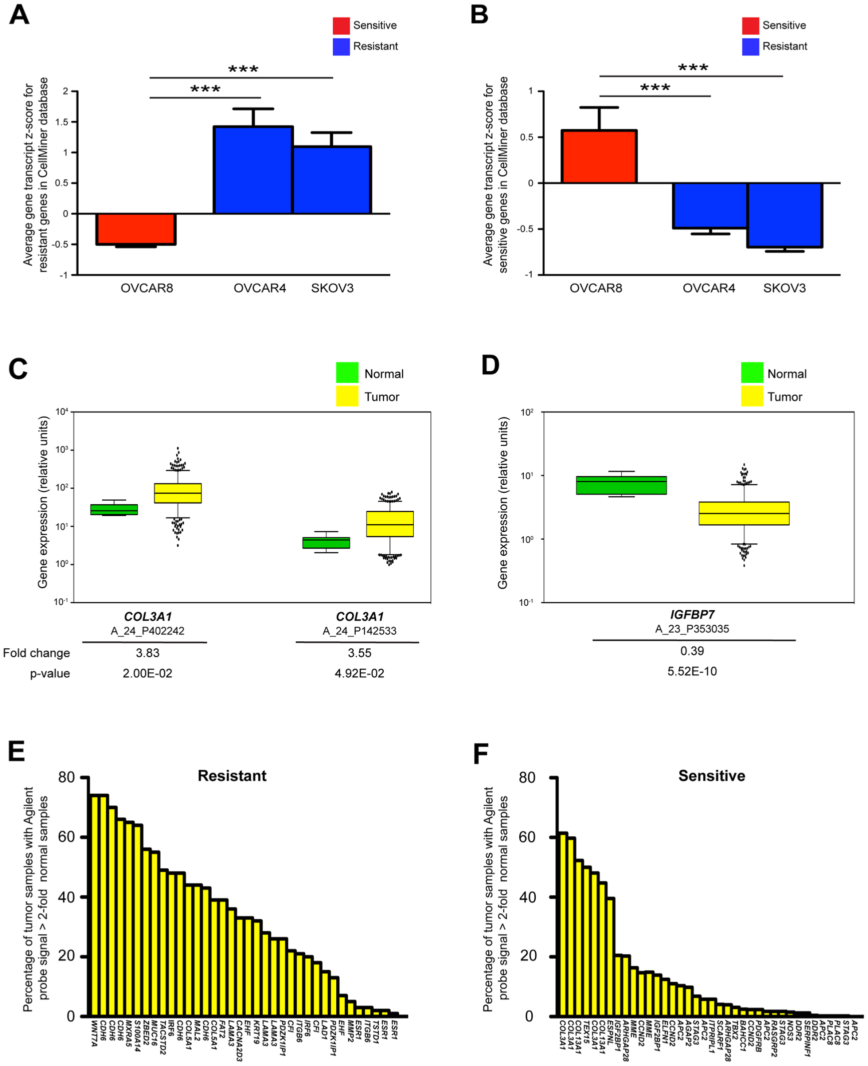

Supplementary Figure 9: Investigation of publicly available datasets for prevalence of genetic signatures. **(A, B)** Average gene transcript z-score from the CellMiner dataset for resistant and sensitive genes from OVCAR8, OVCAR4 and SKOV3 cell lines. Data are displayed as bar graphs representing average z-scores ± standard error of the mean. Positive and negative columns signify higher and lower z-scores, respectively. *** = $p < 0.001$. **(C)** Average expression values of Agilent probes related to an increase in the sensitive signature from 518 TCGA tumors relative to 8 fallopian control samples. Data are displayed as box plots representing 1 gene (derived from 2 unique probes) that showed average increase of expression in tumors (yellow) greater than 2-fold increase over fallopian (green). **(D)** Average expression values of Agilent probes related to a decrease in the resistant signature from 518 TCGA tumors relative to 8 fallopian control samples. Data is displayed as box plots representing 1 gene (derived from 1 unique probe) that showed average decrease of expression in tumors (yellow) greater than 2-fold increase over fallopian (green). The whiskers of each box plot represent expression values at 5th and 95th percentiles. Average fold change expression and p-values are indicated for each probe. **(E, F)** Percentage of 518 tumors in the ovarian TCGA dataset that express individual Agilent probes for the resistant and sensitive signature, respectively. Data are displayed as the percentage of tumors exhibiting single probe expression levels > 2-fold fallopian tissue for the same probe.

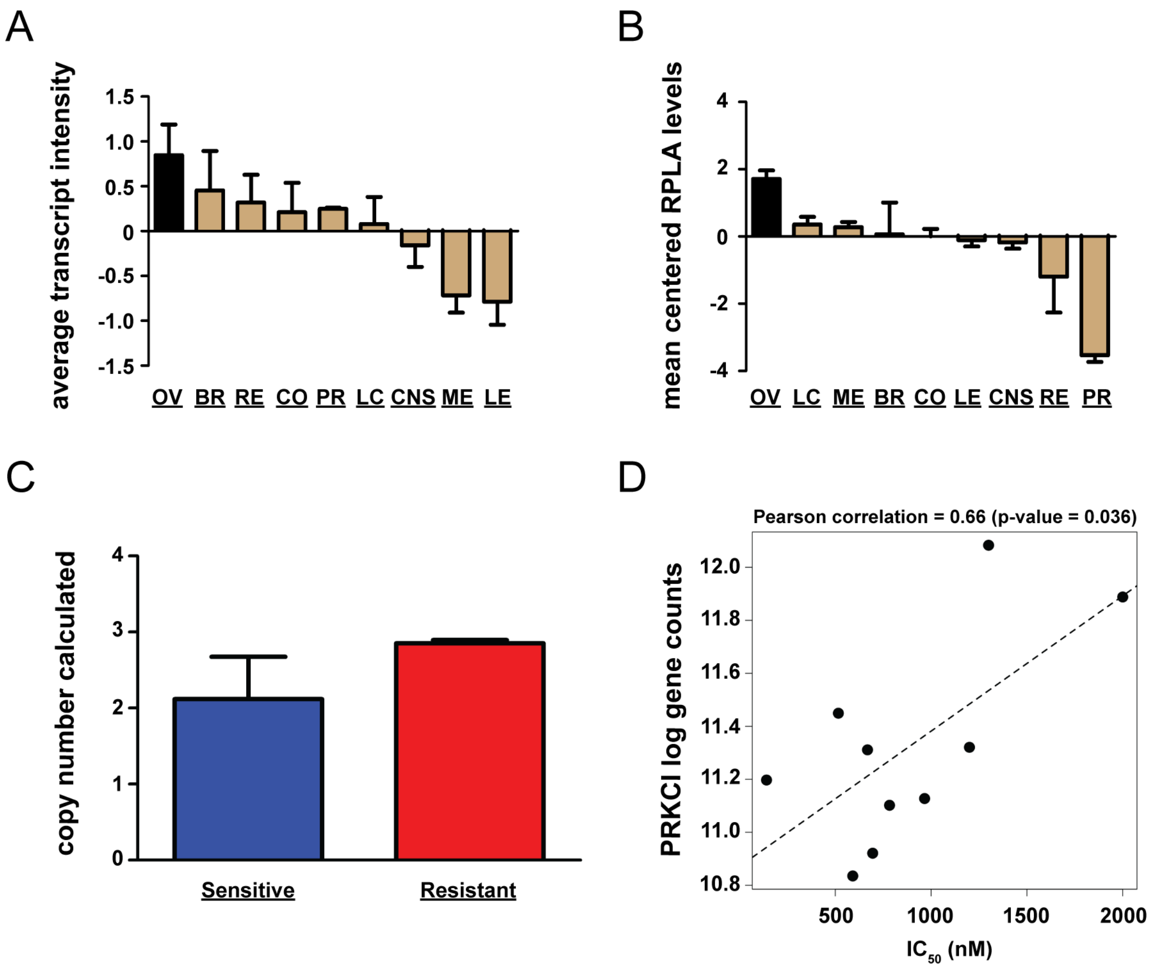

Supplementary Figure 10: PRKCI expression and copy number analysis using ovarian cell line models. **(A, B)** Average gene transcript z-score and protein RPLA levels from the CellMiner dataset for all lines within nine different human cancer types, respectively. Data are displayed as bar graphs representing average scores ± standard error of the mean. Positive and negative columns signify higher and lower z-scores. OV-ovarian cancer, BR-breast cancer, RE-renal cancer, CO-colon cancer, PR-prostate cancer, LC-non small cell lung cancer, CNS-central nervous system, ME-melanoma, LE-leukemia. **(C)** Calculated copy number values for the sensitive (A1847, A2789, OVCAR8) and resistant (OVCAR4, PEO4, SKOV3) cell lines using CopyCaller® software. Data are displayed as bar graphs representing average scores ± standard error of the mean. **(D)** PRKCI log gene counts versus IC_{50} values of ten ovarian cell lines treated with auranofin. A Pearson coefficient was determined to measure linear correlation between the two variables, p-value < 0.05 is considered significant.

Supplementary Table 1 : List of cell lines and compounds used in preliminary high-throughput drug screen. Panel of 10 EOC cell lines utilized in a high-throughput drug screen encompassing 7 FDA-approved compounds and 12 different two-drug combinations.

Cell line	Description
**A1847**	untreated ovarian tumor
**A2780**	untreated ovarian tumor
**C30**	A2780 after intermittent exposure to cisplatin
**CP70**	A2780 after intermittent exposure to cisplatin
**OVCAR4**	ovarian tumor from a patient refractory to cisplatin
**OVCAR5**	untreated ovarian tumor (advanced)
**OVCAR8**	ovarian tumor from a carboplatin-refractory patient
**OVCAR10**	ovarian tumor from a cisplatin/carboplatin refractory patient
**PEO4**	ovarian tumor after patient became refractory
**SKOV3**	adenocarcinoma
**Compound**	**Mechanism**
**Auranofin**	reactive oxygen species inducer
**Elesclomol**	reactive oxygen species inducer
**Bortezomib**	26S proteasome inhibitor
**Carfilzomib**	20S proteasome inhibitor
**Geldanamycin**	ATP-binding pocket inhibitor
**Ganetespib**	ATP-binding pocket inhibitor
**AUY922**	Atp-binding pocket inhibitor

Supplementary Table 2 : Quantification of isoforms utilized in construction of the resistant signature. Expression of variant transcripts for genes related to resistance were compiled and quantified for the six cell lines. Data also display total average number of transcripts detected for each cell line as well as group averages per RefSeq ID.

Gene ID	RefSeq ID	A1847	A2780	OVCAR8	Average	OVCAR4	PEO4	SKOV3	Average
**CDH6**	NM_004932.3	8	12	2	**7**	9361	3567	7167	**6698**
**IGFBP7**	NM_001253835.1	0	0	1	**0**	22	6	34	**21**
**IGFBP7**	NM_001553.2	0	0	0	**0**	2695	494	3637	**2275**
**MXRA5**	NM_015419.3	0	0	0	**0**	2682	243	1957	**1627**
**ITGB6**	NM_000888.3	0	1	1	**1**	1075	2005	431	**1170**
**WNT7A**	NM_004625.3	0	1	0	**0**	2845	213	1338	**1465**
**TACSTD2**	NM_002353.2	24	0	7	**10**	5501	14808	2020	**7443**
**KRT19**	NM_002276.4	9	22	3	**11**	9757	9349	350	**6485**
**ESR1**	NM_000125.3	0	0	0	**0**	0	188	659	**282**
**ESR1**	NM_001122740.1	0	0	0	**0**	0	618	121	**246**
**ESR1**	NM_001122741.1	0	0	0	**0**	36	1039	493	**523**
**ESR1**	NM_001122742.1	0	0	0	**0**	0	0	0	**0**
**MUC16**	NM_024690.2	1	4	3	**3**	9897	14583	28	**8169**
**EHF**	NM_001206615.1	0	0	1	**0**	45	19	11	**25**
**EHF**	NM_001206616.1	0	0	0	**0**	0	0	72	**24**
**EHF**	NM_012153.5	0	1	0	**0**	996	354	198	**516**
**S100A14**	NM_020672.2	3	3	5	**4**	3336	3422	25	**2261**
**MAL2**	NM_052886.2	41	4	15	**20**	5604	15787	1066	**7486**
**LAMA3**	NM_000227.3	28	0	6	**11**	12834	7880	1347	**7354**
**LAMA3**	NM_001127717.1	0	0	0	**0**	0	0	0	**0**
**LAMA3**	NM_001127718.1	0	1	0	**0**	0	0	0	**0**
**LAMA3**	NM_198129.1	19	0	49	**23**	425	218	472	**371**
**ZBED2**	NM_024508.4	1	0	1	**1**	79	717	642	**479**
**LAD1**	NM_005558.3	1	9	0	**3**	4063	978	37	**1693**
**IRF6**	NM_001206696.1	0	1	0	**0**	192	34	0	**76**
**IRF6**	NM_006147.3	0	0	0	**0**	653	307	47	**335**
**CFI**	NM_000204.3	1	0	1	**1**	72	1159	191	**474**
**CACNA2D3**	NM_018398.2	0	0	0	**0**	209	64	144	**139**
**TSTD1**	NM_001113205.1	0	0	0	**0**	46	8	2	**18**
**TSTD1**	NM_001113206.1	0	0	0	**0**	96	31	19	**49**
**TSTD1**	NM_001113207.1	0	0	0	**0**	230	72	27	**110**
**COL5A1**	NM_000093.3	3	34	13	**17**	62	1053	23741	**8285**
**MMP2**	NM_001127891.1	0	4	0	**1**	29	0	0	**10**

(*Continued*)

Gene ID	RefSeq ID	A1847	A2780	OVCAR8	Average	OVCAR4	PEO4	SKOV3	Average
**MMP2**	NM_004530.4	0	0	2	**1**	0	796	1153	**650**
**PDZK1IP1**	NM_005764.3	0	0	1	**0**	79	247	227	**184**
**FAT2**	NM_001447.2	22	20	10	**17**	11481	523	35	**4013**
**Average**		4	3	3		2345	2244	1325	

Supplementary Table 3 : Quantification of isoforms utilized in construction of the sensitive signature. Expression of variant transcripts for genes related to sensitivity were compiled and quantified for the six cell lines. Data also display total average number of transcripts detected for each cell line as well as group averages per RefSeq ID.

Gene ID	RefSeq ID	A1847	A2780	OVCAR8	Average	OVCAR4	PEO4	SKOV3	Average
**AGAP2**	NM_001122772.2	0	381	0	**127**	0	0	0	**0**
**AGAP2**	NM_014770.3	43	429	69	**181**	3	0	5	**3**
**APC2**	NM_005883.2	196	878	33	**369**	4	4	10	**6**
**ARHGAP28**	NM_001010000.2	147	2806	396	**1116**	1	21	18	**13**
**BAHCC1**	NM_001080519.2	435	2381	53	**956**	3	3	37	**14**
**CCND2**	NM_001759.3	15	18074	18	**6036**	3	1	2	**2**
**COL13A1**	NM_001130103.1	0	0	0	**0**	0	0	0	**0**
**COL13A1**	NM_080798.3	6	0	206	**71**	5	0	0	**2**
**COL13A1**	NM_080800.3	38	0	257	**98**	0	0	0	**0**
**COL13A1**	NM_080801.3	20	17	36	**24**	0	0	0	**0**
**COL13A1**	NM_080802.3	33	0	307	**113**	0	0	0	**0**
**COL13A1**	NM_080805.3	64	0	148	**71**	0	5	7	**4**
**COL3A1**	NM_000090.3	11	23906	63	**7993**	4	132	12	**49**
**DDR2**	NM_001014796.1	0	318	0	**106**	0	0	0	**0**
**DDR2**	NM_006182.2	24	4231	46	**1434**	13	9	37	**20**
**ELFN1**	NM_001128636.2	105	2177	77	**786**	2	0	0	**1**
**ESPNL**	NM_194312.2	150	371	19	**180**	1	5	1	**2**
**FREM2**	NM_207361.4	229	4143	192	**1521**	2	48	1	**17**
**IGF2BP1**	NM_001160423.1	0	27	0	**9**	0	3	0	**1**
**IGF2BP1**	NM_006546.3	3623	14860	152	**6212**	25	0	2	**9**
**ITPRIPL1**	NM_001008949.2	150	172	106	**143**	0	0	0	**0**
**ITPRIPL1**	NM_001163523.1	24	310	69	**134**	1	0	0	**0**
**ITPRIPL1**	NM_001163524.1	35	1	0	**12**	0	0	1	**0**
**ITPRIPL1**	NM_178495.5	12	0	0	**4**	0	0	0	**0**
**MME**	NM_000902.3	122	26	147	**98**	0	9	0	**3**
**MME**	NM_007287.2	279	0	216	**165**	2	0	8	**3**
**MME**	NM_007288.2	27	0	0	**9**	0	0	0	**0**
**MME**	NM_007289.2	45	0	0	**15**	0	0	0	**0**

(*Continued*)

Gene ID	RefSeq ID	A1847	A2780	OVCAR8	Average	OVCAR4	PEO4	SKOV3	Average
**NOS3**	NM_000603.4	222	858	277	**452**	8	11	19	**13**
**NOS3**	NM_001160109.1	0	0	0	**0**	0	0	0	**0**
**NOS3**	NM_001160110.1	0	2	0	**1**	0	0	0	**0**
**NOS3**	NM_001160111.1	2	0	0	**1**	0	0	0	**0**
**PDGFRB**	NM_002609.3	422	676	334	**477**	1	1	39	**14**
**PLAC8**	NM_001130715.1	2	0	0	**1**	0	0	0	**0**
**PLAC8**	NM_001130716.1	65	0	37	**34**	41	0	21	**20**
**PLAC8**	NM_016619.2	3626	59	3231	**2306**	0	13	47	**20**
**RASGRP2**	NM_001098670.1	0	60	0	**20**	0	1	0	**0**
**RASGRP2**	NM_001098671.1	44	37	0	**27**	0	0	0	**0**
**RASGRP2**	NM_153819.1	0	83	33	**39**	0	0	0	**0**
**SCARF1**	NM_003693.2	21	13	307	**114**	0	0	0	**0**
**SCARF1**	NM_145350.1	0	0	35	**12**	0	0	0	**0**
**SCARF1**	NM_145352.2	17	7	0	**8**	2	6	1	**3**
**SCARF1**	NR_028075.1	0	0	0	**0**	0	0	0	**0**
**SCARF1**	NR_028076.1	13	6	32	**17**	0	0	0	**0**
**SERPINF1**	NM_002615.5	25	2867	39	**977**	5	3	2	**3**
**STAG3**	NM_012447.2	2811	34	1	**948**	23	7	6	**12**
**TBX2**	NM_005994.3	42	11520	65	**3876**	16	10	37	**21**
**TEX15**	NM_031271.3	72	3782	38	**1297**	1	14	1	**5**
**Average**		275	1990	147		3	6	7	

Supplementary Table 4 : List of lncRNAs upregulated in sensitive and resistant panels. lncRNA sequencing analyses was condensed to Gene ID, Gene name, logFC (log2-fold change) and FDR (Benjamini-Hochberg adjusted p-value to account for multiple comparisons).The lists of lncRNAs that exhibit the highest logFC and FDR in the three sensitive cell lines as well as those from the three resistant lines are shown

**SENSITIVE LINES**			
**Gene ID**	**Gene name**	**logFC**	**FDR**
**HOTAIR**	HOX transcript antisense RNA	8.4	0.013
**LINC02381**	Long intergenic non-protein coding RNA 2381	5.9	0.039
**AFAP1-AS1**	AFAP1 antisense RNA 1	6.8	0.039

**RESISTANT LINES**			
**Gene ID**	**Gene name**	**logFC**	**FDR**
**LINC02167**	Long intergenic non-protein coding RNA 2167	11.7	0.005
**EMX2OS**	EMX2 opposite strand/ antisense RNA	7.7	0.007

(***Continued***)

RESISTANT LINES			
**Gene ID**	**Gene name**	**logFC**	**FDR**
**LINC01559**	Long intergenic non-protein coding RNA 1559	9.2	0.013
**FLJ22447**	Uncharacterized LOC400221	5.3	0.013
**ZNF625-ZNF20**	ZNF625-ZNF20 readthrough (NMD candidate)	7.5	0.024
**C8orf34-AS1**	C8orf34 antisense RNA 1	9.3	0.039

Supplementary Table 5 : List of cycle threshold variations of the resistant genetic signature related to auranofin and AUY922. Absolute cycle threshold variation of 23 genes from the resistant signature in A1847 and PEO4 cultured cells treated with the indicated compounds, both alone and in combination (Aur + AUY). Data represent the difference of absolute threshold cycle ± standard error of the mean as compared to basal transcription from triplicate reactions of two independent experiments. Positive and negative numbers signify higher and lower Ct values, respectively

Gene ID	Auranofin A1847	Auranofin PEO4	AUY922 A1847	AUY922 PEO4	Aur + AUY A1847	Aur + AUY PEO4
**CDH6**	-0.7	1.2	-1.1	-0.2	-0.7	0.1
**IGFBP7**	NA	0.3	NA	0.1	NA	0.6
**MXRA5**	NA	0.9	NA	-0.1	NA	0.4
**ITGB6**	-0.2	0.3	1.6	0.6	0.9	1.0
**WNT7A**	NA	0.8	NA	0.3	NA	2.1
**TACSTD2**	-1.5	0.6	0.3	0.3	-0.8	1.4
**KRT19**	-1.6	0.4	-0.8	0.1	0.6	0.3
**ESR1**	NA	1.0	NA	0.0	NA	0.4
**MUC16**	1.2	-0.4	2.6	-0.2	1.0	0.2
**EHF**	-2.0	-0.2	-1.4	-0.1	-1.5	0.0
**S100A14**	-0.2	0.7	0.0	-0.2	-0.8	-0.1
**MAL2**	-0.7	1.3	-1.2	-0.1	-1.6	0.1
**LAMA3**	0.3	0.3	-0.5	0.7	1.3	0.9
**ZBED2**	3.3	0.2	-1.0	-0.1	1.3	0.3
**LAD1**	-2.1	0.6	-2.7	0.3	-1.6	0.3
**IRF6**	1.1	0.7	0.5	-0.2	1.3	0.1
**CFI**	0.3	1.2	0.2	0.2	-0.3	0.1
**CACNA2D3**	NA	0.5	NA	0.0	NA	0.7
**TSTD1**	> -15.7	0.8	> -15.9	-0.2	> -16.5	0.1
**COL5A1**	NA	0.6	NA	0.0	> -17.2	0.5
**MMP2**	NA	0.6	NA	0.0	NA	0.5
**PDZK1IP1**	1.1	0.0	-1.0	-0.6	-0.5	-0.1
**FAT2**	0.7	0.3	0.1	0.5	1.2	1.4

Supplementary Table 6: List of Agilent probes from the ovarian TCGA tumor dataset demonstrating increased and decreased expression of signature genes as compared to normal tissue. Agilent gene expression data from TCGA on 518 serous adenocarcinomas and 8 fallopian tube samples derived from healthy individuals were queried for the two sets of 23 signature genes. Nine Agilent probes from the resistant signature showed ≥2-fold increase in the average gene expression of its respective genes in the tumor samples relative to controls. Two Agilent probes from the sensitive signature showed ≥2-fold increase in the average gene expression of its respective genes in the tumor samples relative to controls. One Agilent probe from the resistant signature showed ≥2-fold increase in the average gene expression of its respective gene in the control samples relative to tumors. Seven Agilent probes from the sensitive signature showed ≥2-fold increase in the average gene expression of its respective gene in the control samples relative to tumors. Data from multiple probes shown when available. The p-values were calculated using an unpaired two-tailed t-test using GraphPad Prism.

**Increased expression of Resistant signature genes in TCGA tumor samples**							
**Gene ID**	**Agilent Probe ID**	**Normal Expression**	**SEM**	**Tumor Expression**	**SEM**	**Fold Change**	**p value**
**CDH6**	A_24_P687	2.41	0.28	7.242	0.21	3.00	3.85E-03
**IRF6**	A_23_P748	5.36	0.51	11.417	0.26	2.13	4.53E-03
**MXRA5**	A_23_P258136	2.25	0.48	7.560	0.24	3.36	6.08E-03
**WNT7A**	A_23_P258410	1.74	0.14	12.346	0.52	7.08	1.21E-02
**MUC16**	A_23_P5211	3.98	0.86	10.417	0.33	2.62	1.59E-02
**CDH6**	A_23_P214011	4.76	0.78	29.476	1.32	6.19	2.02E-02
**MAL2**	A_23_P60130	3.30	0.24	7.050	0.20	2.13	2.21E-02
**ZBED2**	A_23_P113793	25.76	4.32	73.089	2.74	2.84	3.24E-02
**S100A14**	A_23_P124619	0.63	0.10	2.377	0.11	3.80	4.97E-02
**Decreased expression of Resistant signature genes in TCGA tumor samples**							
**Gene ID**	**Agilent Probe ID**	**Normal Expression**	**SEM**	**Tumor Expression**	**SEM**	**Fold Change**	**p value**
**IGFBP7**	A_23_P353035	7.75	0.87	3.05	0.09	0.39	5.52E-10
**Increased expression of Sensitive signature genes in TCGA tumor samples**							
**Gene ID**	**Agilent Probe ID**	**Normal Expression**	**SEM**	**Tumor Expression**	**SEM**	**Fold Change**	**p value**
**COL3A1**	A_24_P402242	4.24	0.59	16.28	0.64	3.83	0.02
**COL3A1**	A_23_P142533	29.51	3.69	104.83	4.80	3.55	0.05
**Decreased expression of Sensitive signature genes in TCGA tumor samples**							
**Gene ID**	**Agilent Probe ID**	**Normal Expression**	**SEM**	**Tumor Expression**	**SEM**	**Fold Change**	**p value**
**PLAC8**	A_24_P183128	1.43	0.25	0.16	0.01	0.11	8.41E-33
**STAG3**	A_23_P145657	3.27	0.51	0.58	0.03	3.55	5.36E-27
**DDR2**	A_32_P88965	1.19	0.24	0.53	0.02	0.44	3.40E-05
**SERPINF1**	A_23_P100660	1.67	0.26	0.76	0.03	0.45	4.63E-04
**BAHCC1**	A_23_P49539	0.53	0.04	0.25	0.01	0.47	0.003
**CCND2**	A_24_P278747	1.46	0.12	0.59	0.03	0.41	0.001
**TBX2**	A_23_P164451	2.66	0.27	1.13	0.07	0.43	0.01
